# Supplementation with whey peptide rich in β-lactolin improves trait anxiety and subjective stress in healthy adults: a randomized, double-blind, placebo-controlled study

**DOI:** 10.1038/s41598-024-73780-3

**Published:** 2024-10-08

**Authors:** Tatsuhiro Ayabe, Masakazu Shinohara, Masahiro Kita, Chika Takahashi, Jiro Saito, Tomoyuki Furuyashiki, Kenji Toba, Satoshi Umeda, Yasuhisa Ano

**Affiliations:** 1 KIRIN Central Research Institute, Kirin Holdings Company, Limited, Fujisawa, Japan; 2 Institute of Health Sciences, Kirin Holdings Company, Limited, 26-1, Muraoka-Higashi 2-chome, Fujisawa-shi, Fujisawa, 251-8555 Japan; 3 Division of Molecular Epidemiology, Kobe University Graduate School of Medicine, Kobe, Japan; 4 The Integrated Center for Mass Spectrometry, Kobe University Graduate School of Medicine, Kobe, Japan; 5 Medical Station Clinic, Takaban, Meguro-ku, Tokyo, Japan; 6 Division of Pharmacology, Kobe University Graduate School of Medicine, Kobe, Japan; 7 National Center for Geriatrics and Gerontology, Tokyo, Japan; 8 Department of Psychology, Keio University, Tokyo, Japan

**Keywords:** β-Lactolin, Mental disorders, Quality of life, Immunologic response, Anxiety, Salivary stress markers, Nutritional supplements, Psychology

## Abstract

Mental disorders have become one of the most burdensome health concerns. We have previously demonstrated that whey-derived β-lactolin (glycine–thereonine–tryptophan–tyrosine tetrapeptide) activates dopaminergic systems and improves psychiatric function in rodents. However, the effects of β-lactolin on human mood states have not been investigated. This randomized, double-blind, placebo-controlled study aimed to evaluate the effects of supplementation with β-lactolin–rich whey peptide on human mood states. Sixty healthy adults (aged 45–64 years) with relatively low psychological health were randomly allocated to receive either whey peptide (containing β-lactolin 1.6 mg/day) or placebo for 6 weeks. Mood states (primary outcomes) were evaluated using self-reporting questionnaires. Health-related quality of life (QOL), salivary stress marker and lipid mediator levels were evaluated as secondary outcomes. Compared with placebo, supplementation with β-lactolin improved changes in trait anxiety (*p* = 0.046), as assessed using the state–trait anxiety inventory, and in subjective stress (*p* = 0.043), as assessed using the Perceived Stress Scale. In the assessment of QOL, changes in the vitality subscale and mental health summary score of the 36-Item Short-Form Health Survey were improved in the β-lactolin group. The levels of salivary immunoglobulin A were significantly higher in the β-lactolin group. In a subgroup analysis by median age (54.5 years), subjective stress and salivary prostaglandin levels were significantly decreased by β-lactolin supplementation in the 45–54 -year-old subgroup. In conclusion, supplementation with β-lactolin improves trait anxiety, subjective stress, and psychological QOL, which may be associated with immunologic responses detected via salivary analysis.

**Supplementary Information:**

The online version contains supplementary material available at 10.1038/s41598-024-73780-3.

## Introduction

Mental disorders are one of the most burdensome health concerns in our rapidly growing society. In 2019, the World Health Organization reported that 970 million people worldwide were affected by mental disorders, among which depressive disorders and anxiety disorders are commonly included^1^. Furthermore, the worldwide outbreak of COVID-19 beginning in 2020 had a serious influence on the mental health of the population^2,3^. Although standard treatments such as pharmacotherapy and psychotherapy have been established, primary prevention before the onset of mental disorders in daily life is important. Indeed, previous studies have reported that primary prevention, such as cognitive behavioral therapy, preventive psychopharmacologic treatment, daily exercise, and following a healthy diet, shows efficacy in preventing mood disorders^4,5^. Healthy dietary habits or nutritional interventions are important factors in primary prevention; however, evidence-based solutions for nutrition have not been fully established.

Several systematic reviews and meta-analyses of epidemiological studies have proposed that specific dietary patterns are effective for preventing mental disorders^6,7^. A recent meta-analysis of cohort studies revealed that the consumption of fermented dairy products was associated with decreased depression risk, as assessed by self-report questionnaires^8^. A cross-sectional study of Japanese adults reported that a higher frequency of low-fat dairy consumption, but not whole-fat dairy, was associated with a lower prevalence of depressive symptoms^9^. In fermented dairy products, a variety of fatty acids or peptides are released during the fermentation process, and these compounds have been shown to exhibit various functions, including psychiatric functions. A milk-derived tyrosine–leucine–glycine tripeptide and its core structure tyrosine–leucine, for example, have shown anxiolytic and antidepressant effects, and these activities were demonstrated to be comparable with those of the preexisting anxiolytic diazepam^10,11^. Although several bioactive peptides are expected to prevent depression or anxiety via monoamine- and GABA-related mechanisms based on preclinical studies, there remains a lack of clinical evidence.

We have previously identified tryptophan–tyrosine (WY)–related peptides including glycine–threonine–tryptophan–tyrosine, designated as β-lactolin, released from β-lactoglobulin in whey protein^12,13^. WY-related peptides, including β-lactolin, are abundant in fermented dairy products such as camembert cheese and are richly released from whey protein digestion with specific enzymes. We have demonstrated that WY-related peptides improve spatial working and episodic memory via the inhibition of monoamine oxidase-B and the subsequent increase in dopamine levels in the brain and activation of dopamine D1-like receptors in rodents^12,14^. The administration of β-lactolin also improved depression-like behavior of rodents, as assessed in the tail suspension test, which was inhibited by the inhibition of dopamine D1-like receptor^15^. We have also shown that, as compared with placebo, the supplementation with whey peptides richly containing β-lactolin (1.6 mg/day) for 6 and 12 weeks improved memory, attention, and executive functions associated with the function of the dorsolateral prefrontal cortex in middle-aged adults (45–64 years old) with high subjective fatigue^16^. In another clinical trial, researchers showed that supplementation with β-lactolin–rich whey peptide in healthy subjects aged 50–75 years for 12 weeks enhanced cognitive performance, such as associative learning memory and control of attention, as compared with the placebo-controlled group^17^. Furthermore, β-lactolin was reported to improve both cerebral blood flow (CBF) in the dorsolateral prefrontal cortex and event-related P300 amplitude during cognitive tasks^18–20^. These studies demonstrate that supplementation with β-lactolin in middle- or older-aged adults improves CBF, neuronal activity, and cognitive functions. Serial preclinical and clinical studies led us to assume that β-lactolin supplementation improves human mood states such as depression and anxiety. However, the effects of β-lactolin on human mood states have not been investigated to date.

We conducted a randomized, double-blind, placebo-controlled clinical trial to identify the effects of β-lactolin on mood states of healthy adult subjects using questionnaire-based mood state assessments. Health-related quality of life (QOL) was also investigated. Furthermore, to explore the underlying mechanisms of β-lactolin function, we evaluated the levels of salivary stress-related markers and lipid mediators. This is the first clinical trial to evaluate the effect of whey-derived peptides on humane mood states.

## Results

### Subject characteristics

**Fig. 1:**
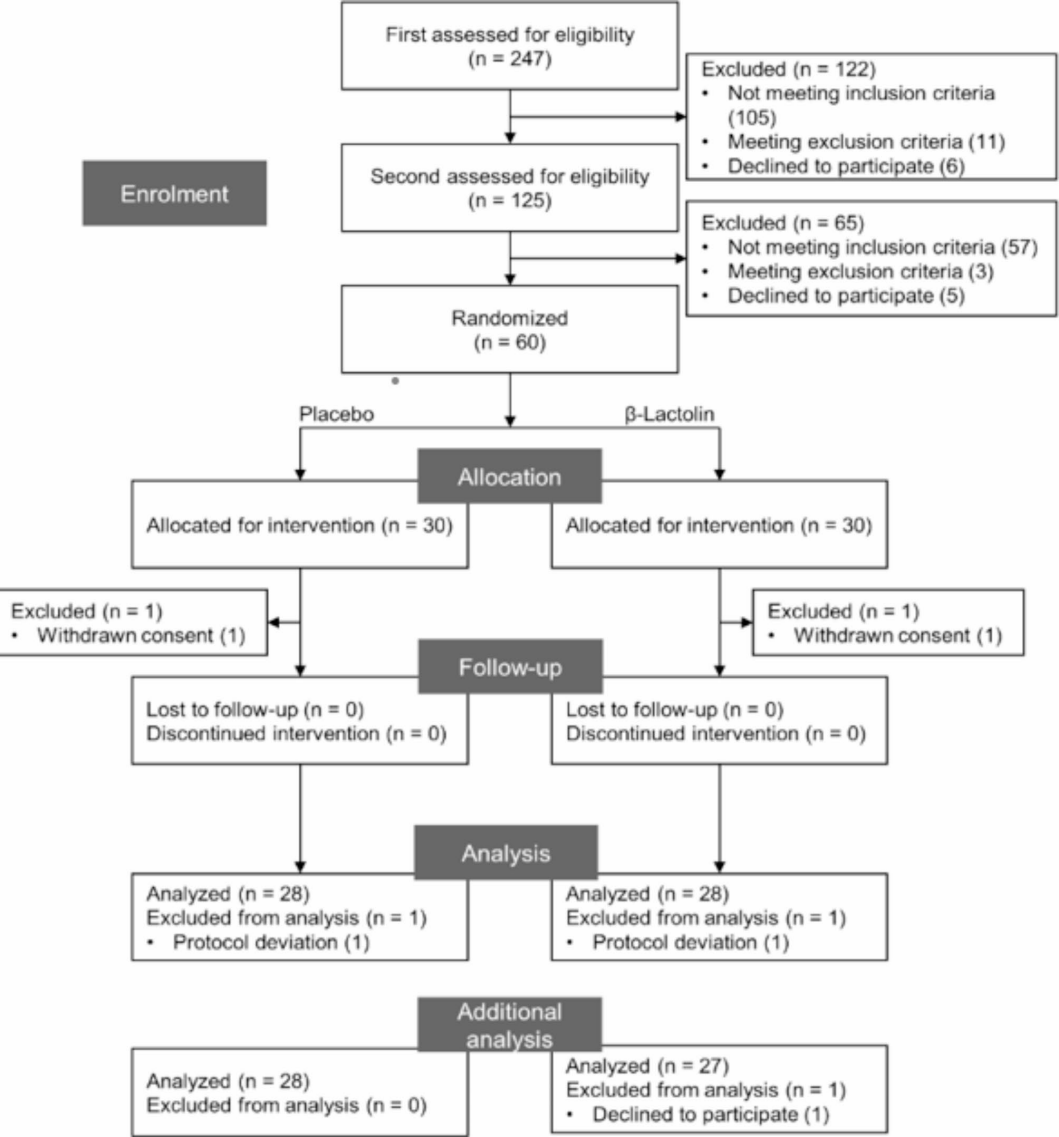
CONSORT diagram. A total of 247 subjects were screened, and 60 subjects were included in the study. Subjects were randomly allocated to the β-lactolin (*n* = 30) or placebo (*n* = 30) groups. Each subject in both the β-lactolin and placebo group declined their consent. One subject in the β-lactolin group and one subject in the placebo group were excluded from the analysis due to deviation from the protocol, leaving 28 subjects for analysis in each group. In the additional analysis, one subject in the β-lactolin group declined to participate.

A flowchart of the subject selection process is presented in Fig. 1. We screened 247 subjects in the first screening period; 125 subjects proceeded to the second screening period, and 122 subjects were excluded due to not feeling daily life annoying or anxiety (*n* = 2), Beck Depression Inventory–Second Edition (BDI–II) scores > 19 (*n* = 89), relatively low scores (< 2) on the General Health Questionnaire–28 (GHQ-28; *n* = 14), high habitual consumption of alcohol (*n* = 3), sleep disorders (*n* = 1), intake of antibiotics within the past 3 months (*n* = 1), unsuitable lifestyle (*n* = 1), or determined by the principal investigator to be unsuitable for the study (*n* = 5). During the second screening period, 65 subjects were excluded due to the scores of > 19 on the BDI–II (*n* = 22), relatively high variation of mood state assessment scores (*n* = 35), or determined by the principal investigator to be unsuitable for the study (*n* = 3). The remaining 60 subjects were randomly allocated to the β-lactolin or placebo groups and received supplements for 6 weeks. During the intervention, two subjects (β-lactolin group, *n* = 1; placebo group, *n* = 1) were excluded from the study due to withdrawal of participation. Twenty-nine subjects in each group completed the intervention. After data collection, two subjects (β-lactolin group, *n* = 1; placebo group, *n* = 1) were excluded from the analysis due to protocol deviation. Finally, both 28 subjects in each group were analyzed. After the originally designed analysis was completed, we performed additional analysis on the remaining saliva samples. Fifty-five subjects (β-lactolin group, *n* = 27; placebo group, *n* = 28) consented to the additional analysis, and one subject (β-lactolin group) did not. We found no statistically significant differences between the groups in terms of subject characteristics (Table 1).

**Table 1:** Subject characteristics.

Characteristics	Placebo (*n* = 28)	β-lactolin (*n* = 28)	*p* value
Age	53.6 ± 5.5	54.3 ± 4.8	0.605
Male/female	20/8	19/9	1.000
Height (cm)	167.54 ± 8.31	165.40 ± 7.45	0.316
Body weight (kg)	61.70 ± 8.62	60.41 ± 8.96	0.584
Body mass index (kg/m^2^)	21.91 ± 1.87	21.99 ± 2.16	0.880
GHQ score	6.0 ± 2.6	6.5 ± 3.1	0.498

Data are presented as means ± SD. The *p* value shows the between-group difference using unpaired *t* tests for age, height, body weight, body mass index, and GHQ test, using χ^2^ test for male/female.

## Primary outcomes

### Mood state assessments

**Fig. 2:**
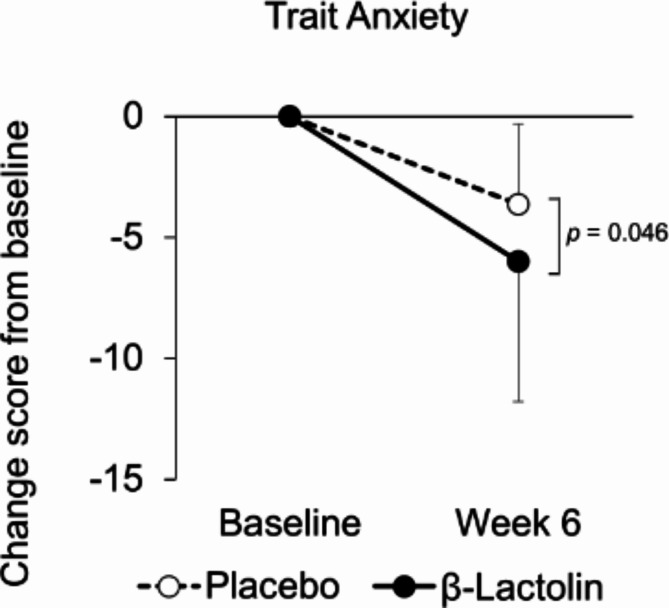
Score changes in STAI trait anxiety. Score changes in trait anxiety from baseline to week 6 were compared between each group. The solid line indicates the β-lactolin group (*n* = 28), and the dotted line indicates the placebo group (*n* = 28). Data are presented as means ± SD. The *p* value shows the between-group difference performed using Mann–Whitney *U* tests.

**Fig. 3:**
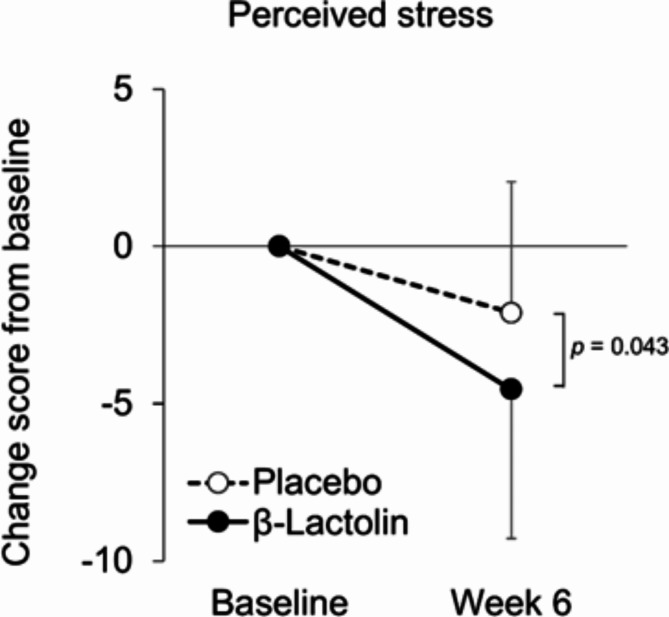
Score changes in perceived stress. Score changes in perceived stress from baseline to week 6 were compared between each group. The solid line indicates the β-lactolin group (*n* = 28), and the dotted line indicates the placebo group (*n* = 28). Data are presented as means ± SD. The *p* value shows the between-group difference performed using Mann–Whitney *U* tests.

Table 2 shows the scores at baseline and at week 6 as well as score changes from baseline to week 6 in mood state assessments (Apathy scale, BDI–II, State–Trait Anxiety Inventory: STAI, Perceived Stress Scale: PSS, Sukemune–Hiew Resilience Test: S-H resilience test, and Five Facet Mindfulness Questionnaire: FFMQ). In the STAI, changes in trait anxiety were significantly decreased in the β-lactolin group as compared with the placebo group (*p* = 0.046; Fig. 2), whereas there were no changes in state anxiety or total score between the groups (*p* = 0.674 and *p* = 0.309, respectively). There was a significant decrease in score changes of the PSS in the β-lactolin group as compared with the placebo group (*p* = 0.043; Fig. 3). There were no differences between the groups in other assessment scores. Although score changes in apathy scale and BDI–II did not change significantly between groups (*p* = 0.104 and *p* = 0.130, respectively), the scores on the apathy scale were significantly decreased in the β-lactolin group at week 6 as compared with baseline (*p* = 0.004), although this change was not observed in the placebo group (*p* = 0.729). These results indicate that the daily intake of β-lactolin improves several aspects of mood state, such as state anxiety and perceived stress.

**Table 2:** Scores and score changes for mood state.

			Baseline (Week 0)	Week 6	Changes from baseline
Apathy scale		Placebo	14.0 ± 6.2	13.8 ± 5.9	-0.3 ± 4.0
		β-lactolin	15.2 ± 4.9	13.3 ± 5.0**	-1.9 ± 3.1
		*p* value	0.286	0.729	0.104
BDI-II		Placebo	11.8 ± 4.3	7.8 ± 4.6**	-4.0 ± 4.2
		β-lactolin	12.4 ± 3.5	6.6 ± 4.8**	-5.8 ± 4.5
		*p* value	0.500	0.244	0.130
STAI	State anxiety	Placebo	42.6 ± 7.6	38.4 ± 6.3**	-4.2 ± 5.9
		β-lactolin	45.0 ± 7.9	40.2 ± 6.7**	-4.8 ± 5.3
		*p* value	0.198	0.283	0.674
	Trait anxiety	Placebo	46.1 ± 7.1	42.5 ± 7.1**	-3.6 ± 3.3
		β-lactolin	47.3 ± 6.9	41.3 ± 7.7**	-6.0 ± 5.8#
		*p* value	0.669	0.305	0.046
	Total	Placebo	88.7 ± 13.2	80.9 ± 11.7**	-7.9 ± 8.1
		β-lactolin	92.2 ± 12.6	81.5 ± 12.9**	-10.8 ± 9.4
		*p* value	0.248	0.848	0.309
PSS		Placebo	39.1 ± 5.1	37.0 ± 5.0*	-2.1 ± 4.2
		β-lactolin	39.5 ± 5.4	34.9 ± 5.0**	-4.5 ± 4.8#
		*p* value	0.741	0.124	0.043
S-H resilience test		Placebo	96.8 ± 13.8	101.3 ± 13.2**	4.5 ± 6.9
		β-lactolin	93.9 ± 11.2	99.9 ± 11.9**	6.0 ± 7.0
		*p* value	0.306	0.385	0.342
FFMQ	Observing	Placebo	21.0 ± 5.2	21.3 ± 5.4	0.4 ± 2.4
		β-lactolin	21.9 ± 5.9	21.4 ± 6.0	-0.5 ± 2.6
		*p* value	0.604	0.99	0.13
	Nonreactivity	Placebo	20.6 ± 3.4	21.9 ± 3.1**	1.4 ± 2.5
		β-lactolin	20.0 ± 3.3	21.4 ± 3.9*	1.4 ± 3.2
		*p* value	0.304	0.57	0.733
	Nonjudging	Placebo	27.2 ± 3.7	27.3 ± 3.4	0.1 ± 2.9
		β-lactolin	27.2 ± 3.7	28.4 ± 4.4	1.2 ± 3.4
		*p* value	0.964	0.362	0.303
	Describing	Placebo	24.1 ± 5.4	25.2 ± 5.8	1.1 ± 3.0
		β-lactolin	23.9 ± 5.7	24.6 ± 5.3	0.7 ± 2.2
		*p* value	0.742	0.766	0.29
	Acting with Awareness	Placebo	26.7 ± 3.7	27.7 ± 4.1	1.0 ± 3.0
		β-lactolin	27.1 ± 4.1	28.5 ± 4.3*	1.4 ± 3.0
		*p* value	0.772	0.766	0.777

Data are presented as means ± SD. The *p* value shows the between-group difference performed using Mann–Whitney *U* tests. β-lactolin group (*n* = 28); placebo group (*n* = 28); *p < 0.05, **p < 0.01 compared with baseline in each group using paired *t* tests; #*p* < 0.05 in Mann–Whitney *U* tests. BDI–II, Beck Depression Inventory–Second Edition; STAI, State–Trait Anxiety Inventory; PSS, Perceived Stress Scale; FFMQ, Five Facet Mindfulness Questionnaire.

## Secondary outcomes

### Health-related QOL

**Fig. 4:**
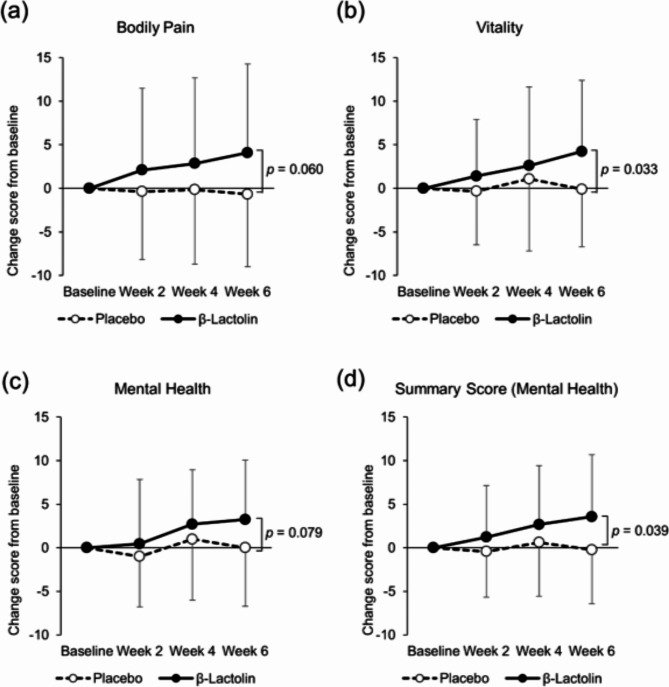
Score changes in the SF-36. Score changes in (**a**) bodily pain, (**b**) vitality, (**c**) mental health subscales, and (**d**) mental health summary score in SF-36 from baseline to weeks 2, 4, and 6 were compared between each group at each time point. The solid line indicates the β-lactolin group (*n* = 28), and the dotted line indicates the placebo group (*n* = 28). Data are presented as means ± SD. The *p* value shows the between-group difference performed using unpaired *t* tests.

Table 3 shows the raw scores at baseline and at weeks 2, 4, and 6 as well as score changes from baseline to weeks 2, 4, and 6 on the 36-Item Short-Form Health Survey (SF-36). Changes in the VT subscale at week 6 were significantly increased in the β-lactolin group as compared with the placebo group (*p* = 0.033; Fig. 4), indicating an improvement in vitality. In the β-lactolin group, changes in the BP and MH subscales at week 6 showed a tendency toward improvement (*p* = 0.060 and *p* = 0.079, respectively). Furthermore, we analyzed two component summary scores for Japanese subjects and found that changes in the psychological summary score (2MCS) were significantly increased in the β-lactolin group compared with the placebo group (*p* = 0.039; Fig. 4).

**Table 3:** Scores and score changes for health-related QOL.

		Score				Changes from baseline		
		Week 0	Week 2	Week 4	Week 6	Week 2	Week 4	Week 6
PF	Placebo	53.02 ± 6.41	53.12 ± 4.84	53.61 ± 6.11	52.73 ± 7.13	0.10 ± 5.33	0.59 ± 5.16	-0.29 ± 6.18
	β-lactolin	51.85 ± 6.03	52.54 ± 6.48	53.03 ± 5.32	53.61 ± 5.69	0.68 ± 4.25	1.18 ± 4.17	1.76 ± 4.59
	p value	0.486	0.703	0.709	0.612	0.653	0.638	0.165
RP	Placebo	52.59 ± 7.13	51.54 ± 7.79	54.74 ± 3.51	52.48 ± 6.30	-1.05 ± 8.75	2.15 ± 5.91	-0.10 ± 7.30
	β-lactolin	51.24 ± 9.44	53.10 ± 4.33	53.50 ± 5.04	53.93 ± 4.92	1.86 ± 9.70	2.26 ± 9.83	2.69 ± 9.17
	p value	0.55	0.358	0.292	0.344	0.244	0.959	0.213
BP	Placebo	53.08 ± 8.93	52.72 ± 7.39	52.94 ± 8.09	52.41 ± 7.80	-0.36 ± 7.80	-0.15 ± 8.55	-0.67 ± 8.30
	β-lactolin	48.13 ± 10.83†	50.24 ± 6.90	51.00 ± 8.71	52.23 ± 7.68*	2.11 ± 9.40	2.87 ± 9.82	4.10 ± 10.20†
	p value	0.067	0.201	0.392	0.93	0.288	0.226	0.06
GH	Placebo	54.81 ± 7.10	55.04 ± 6.38	56.32 ± 7.11	54.43 ± 8.20	0.23 ± 5.32	1.51 ± 5.97	-0.38 ± 6.51
	β-lactolin	52.61 ± 6.60	53.77 ± 6.93	54.30 ± 7.63	53.98 ± 8.75	1.16 ± 6.26	1.69 ± 7.19	1.37 ± 8.63
	p value	0.235	0.478	0.311	0.842	0.552	0.918	0.396
VT	Placebo	50.49 ± 7.55	50.17 ± 6.79	51.58 ± 7.61	50.39 ± 8.16	-0.32 ± 6.15	1.09 ± 8.28	-0.10 ± 6.58
	β-lactolin	48.75 ± 7.90	50.16 ± 6.37	51.36 ± 8.54	52.99 ± 6.59*	1.41 ± 6.49	2.61 ± 9.02	4.24 ± 8.17#
	p value	0.405	0.997	0.92	0.194	0.312	0.516	0.033
SF	Placebo	53.28 ± 7.08	52.68 ± 6.56	53.28 ± 6.57	53.08 ± 6.86	-0.60 ± 7.72	0.00 ± 5.95	-0.20 ± 6.22
	β-lactolin	50.26 ± 9.32	53.08 ± 6.14	52.87 ± 5.26	53.67 ± 5.51	2.82 ± 9.86	2.61 ± 9.98	3.41 ± 9.96
	p value	0.178	0.815	0.801	0.723	0.154	0.239	0.11
RE	Placebo	51.36 ± 6.75	50.55 ± 8.41	52.41 ± 5.65	52.29 ± 8.48	-0.80 ± 7.99	1.05 ± 5.63	0.93 ± 8.80
	β-lactolin	51.75 ± 6.50	52.29 ± 5.76	53.48 ± 6.10	54.02 ± 4.92	0.53 ± 7.36	1.73 ± 8.55	2.26 ± 7.53
	p value	0.824	0.373	0.498	0.354	0.518	0.729	0.544
MH	Placebo	51.48 ± 7.21	50.48 ± 7.42	52.46 ± 5.88	51.47 ± 6.47	-1.00 ± 5.80	0.99 ± 6.99	0.00 ± 6.70
	β-lactolin	49.49 ± 7.39	49.93 ± 7.45	52.20 ± 7.51*	52.73 ± 6.12*	0.45 ± 7.42	2.71 ± 6.27	3.24 ± 6.85†
	p value	0.312	0.784	0.882	0.458	0.421	0.336	0.079
2PCS	Placebo	53.19 ± 6.48	52.66 ± 6.14	54.37 ± 4.62	53.23 ± 7.64	-0.52 ± 7.09	1.18 ± 6.13	0.05 ± 7.05
	β-lactolin	52.09 ± 7.53	53.58 ± 4.73	53.69 ± 4.53	54.11 ± 3.79	1.49 ± 7.27	1.60 ± 7.69	2.02 ± 7.30
	p value	0.562	0.533	0.579	0.588	0.298	0.824	0.308
2MCS	Placebo	51.70 ± 6.39	51.29 ± 6.12	52.31 ± 5.73	51.48 ± 6.33	-0.42 ± 5.25	0.61 ± 6.19	-0.23 ± 6.22
	β-lactolin	48.97 ± 6.57	50.19 ± 5.98	51.65 ± 7.44*	52.53 ± 6.10*	1.23 ± 5.92	2.68 ± 6.75	3.56 ± 7.13#
	p value	0.12	0.502	0.708	0.53	0.277	0.237	0.039

Data are presented as means ± SD. The *p* value shows the between-group difference performed using unpaired *t* tests. β-lactolin group (*n* = 28); placebo group (*n* = 28); *p < 0.05 compared with baseline in each group using paired *t* tests; #*p* < 0.05 using unpaired *t* tests. PF, physical functioning, RP, role limitations due to physical health; BP, bodily pain; GH, general health perceptions; VT, vitality; SF, social functioning; RE, role limitations due to emotional problems; MH, mental health; PCS, physical component scores; MCS, mental component scores.

### Salivary stress markers

**Fig. 5:**
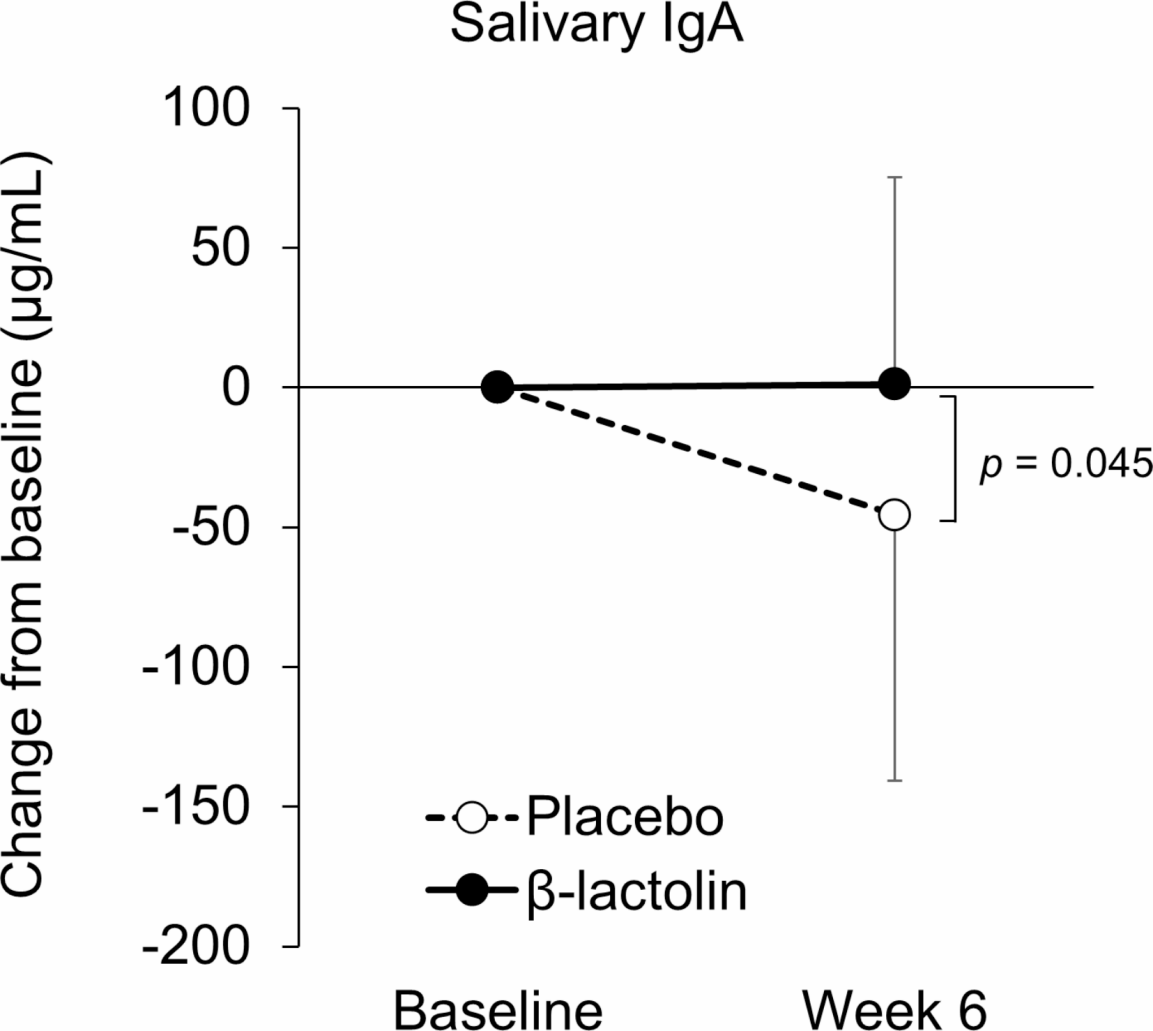
Changes in salivary IgA level. Changes in the salivary IgA level from baseline to week 6 were compared between each group. The solid line indicates the β-lactolin group (*n* = 28), and the dotted line indicates the placebo group (*n* = 28). Data are presented as means ± SD. The *p* value shows the between-group difference using unpaired *t* tests.

To explore the underlying mechanisms of the effects of β-lactolin on anxiety and subjective stress, we measured salivary cortisol, α-amylase, and immunoglobulin A (IgA) levels (Table 4). Changes in IgA levels from baseline to week 6 were significantly higher in the β-lactolin group compared with the placebo group (*p* = 0.045; Fig. 5). In the placebo group, the IgA levels were significantly decreased at week 6 as compared with baseline, which shows that β-lactolin may maintain IgA levels. There were no statistically significant differences in the changes in other stress-related salivary cortisol and α-amylase between the groups.

**Table 4:** Salivary stress marker levels.

		Baseline (week 0)	Week 6	Changes from baseline
IgA	Placebo	184.15 ± 111.28	138.64 ± 70.29*	-45.51 ± 95.35
	β-lactolin	186.04 ± 115.37	187.32 ± 141.99	1.29 ± 74.03#
	p value	0.951	0.11	0.045
Cortisol	Placebo	0.23 ± 0.15	0.20 ± 0.12	-0.03 ± 0.15
	β-lactolin	0.23 ± 0.14	0.21 ± 0.13	-0.02 ± 0.15
	p value	0.945	0.634	0.743
α-amylase	Placebo	109.99 ± 77.72	95.23 ± 63.26	-14.75 ± 45.93
	β-lactolin	115.13 ± 73.80	112.21 ± 70.49	-2.93 ± 27.94
	p value	0.800	0.347	0.250

Data are presented as means ± SD. The *p* value shows the between-group difference performed using unpaired *t* tests. β-lactolin group (*n* = 28); placebo group (*n* = 28); *p < 0.05 compared with baseline in each group using paired *t* tests; #*p* < 0.05 in unpaired *t* tests. IgA, immunoglobulin A.

## Fecal sample analysis

Because the gut microbiome is considered to be deeply connected with mood states, we evaluated fecal short-chain fatty acid (SCFA) concentrations and microorganism composition. We compared changes from baseline to week 6 of the concentration of each SCFA (acetic acid, propionic acid, n-butyric acid, isobutyric acid, n-valenic acid, isovalenic acid, and n-capronic acid) but found no differences between the β-lactolin and placebo group (Supplementary Table 1). Changes from baseline to week 6 in microbiome composition in major phyla (*Firmicutes*, *Actinobacteria*, *Bacteroidetes*, *Proteobacteria*, and *Verrucomicrobia*) and genera (*Bifidobacterium*, *Bacteroides*, *Romboutsia*, *Clostridium*, *Lactobacillus*, and *Lactococcus*) were compared between the groups. There was a significant decrease in the composition changes in the *Actinobacteria* phylum in the β-lactolin group as compared with the placebo group (*p* = 0.016; Supplementary Table 2). The composition of the *Bifidobacterium* genus at baseline was significantly higher in the β-lactolin group; thus, we used analysis of covariance to analyze the changes in *Bifidobacterium* composition and found a significant decrease in the β-lactolin group (*p* = 0.005; Supplementary Table 3).

## Post hoc salivary metabolome analysis

**Fig. 6:**
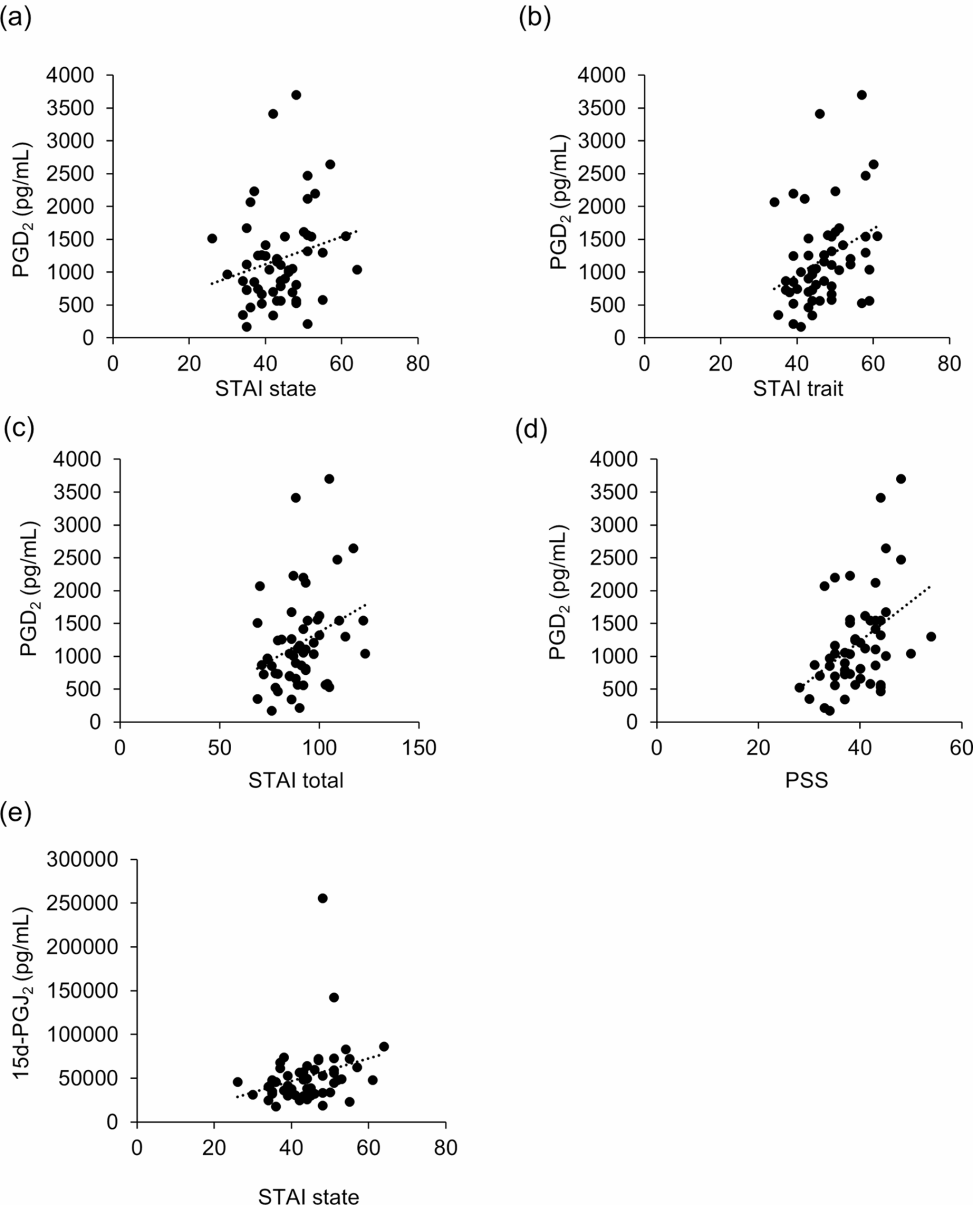
Correlations between salivary prostaglandin levels and mood states. Correlation between baseline salivary prostaglandin levels and mood states were analyzed. The PGD_2_ levels were correlated with (a) STAI state anxiety scores (*r* = 0.287, *p* = 0.034), (b) STAI trait anxiety scores (*r* = 0.373, *p* = 0.005), (c) total scores of STAI (*r* = 0.367, *p* = 0.006), and (d) PSS scores (*r* = 0.390, *p* = 0.003). (e) 15d-PGJ_2_ levels were correlated with STAI state anxiety scores (*r* = 0.369, *p* = 0.006).

**Fig. 7:**
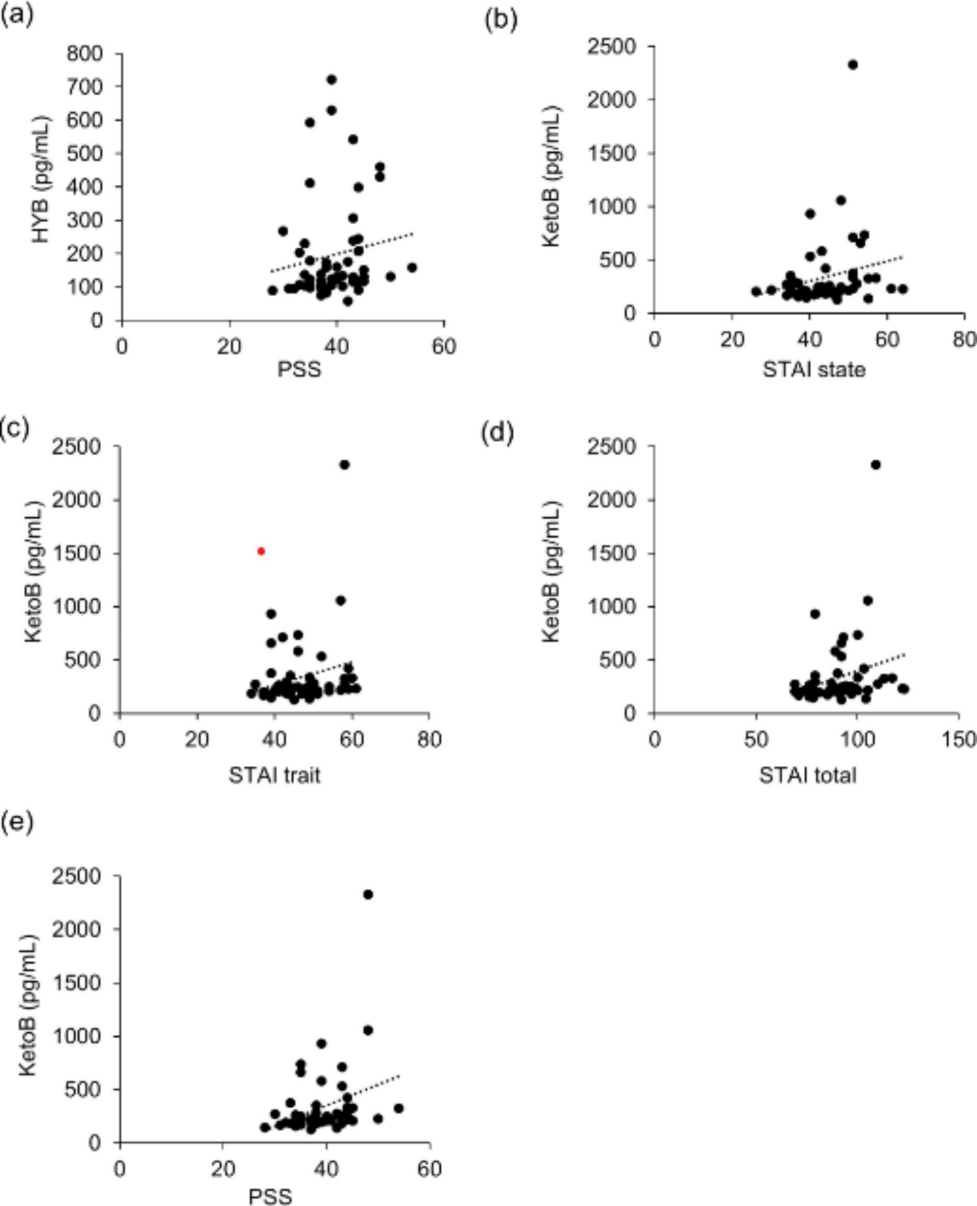
Correlations between salivary linoleic acid metabolite levels and mood states. Correlation between baseline salivary linoleic acid metabolite levels and mood states were analyzed. (**a**) The HYB levels were correlated with PSS scores (*r* = 0.272, *p* = 0.045). Keto B levels were correlated with (**b**) STAI state anxiety scores (*r* = 0.321, *p* = 0.017), (**c**) STAI trait anxiety scores (*r* = 0.272, *p* = 0.045), (**d**) total scores of STAI (*r* = 0.391, *p* = 0.003), and (**e**) PSS scores (*r* = 0.350, *p* = 0.009).

The significant difference in changes in salivary IgA levels led us to further explore the biochemical changes in salivary metabolites. Thus, we performed additional experiments to evaluate lipid mediators (LMs) in the resting saliva samples. The remaining 57 samples with additional informed consent were subjected to LM lipidomics. Before we evaluated the effect of β-lactolin treatment, we explored the relationship between baseline anxiety and subjective stress and LM levels using correlation analysis. The baseline prostaglandin (PG) D_2_ levels were significantly correlated with baseline state and trait anxiety and total scores on the STAI (*r* = 0.287, 0.373, and 0.367, *p* = 0.034, 0.005, and 0.006, respectively) and baseline PSS scores (*r* = 0.390, *p* = 0.003, Fig. 6). The baseline levels of 15-deoxy-delta 12, 14-PGJ_2_ (15d-PGJ_2_), a metabolite of PGD_2_, were also significantly correlated with the baseline state anxiety of STAI (*r* = 0.369, *p* = 0.006). In addition, several linoleic acid metabolites showed a relationship with anxiety and subjective stress. The baseline 10-hydroxy-octadecenoic acid (18:0; HYB) levels were significantly correlated with baseline PSS scores (*r* = 0.272, *p* = 0.045). Baseline 10-oxo-octadecenoic acid (18:0) (Keto B) levels were significantly correlated with baseline state and trait anxiety and total scores of the STAI (*r* = 0.321, 0.272, and 0.391, *p* = 0.017, 0.045, and 0.003, respectively) as well as baseline PSS scores (*r* = 0.350, *p* = 0.009 Fig. 7). Taken together, state and trait anxiety and perceived stress were associated with LM levels.

Next, we compared the changes in salivary LM levels from baseline to week 6 between the placebo and β-lactolin groups, although no statistically significant differences were observed (Supplementary Table 4).

### Subgroup analysis classified by age

**Fig. 8:**
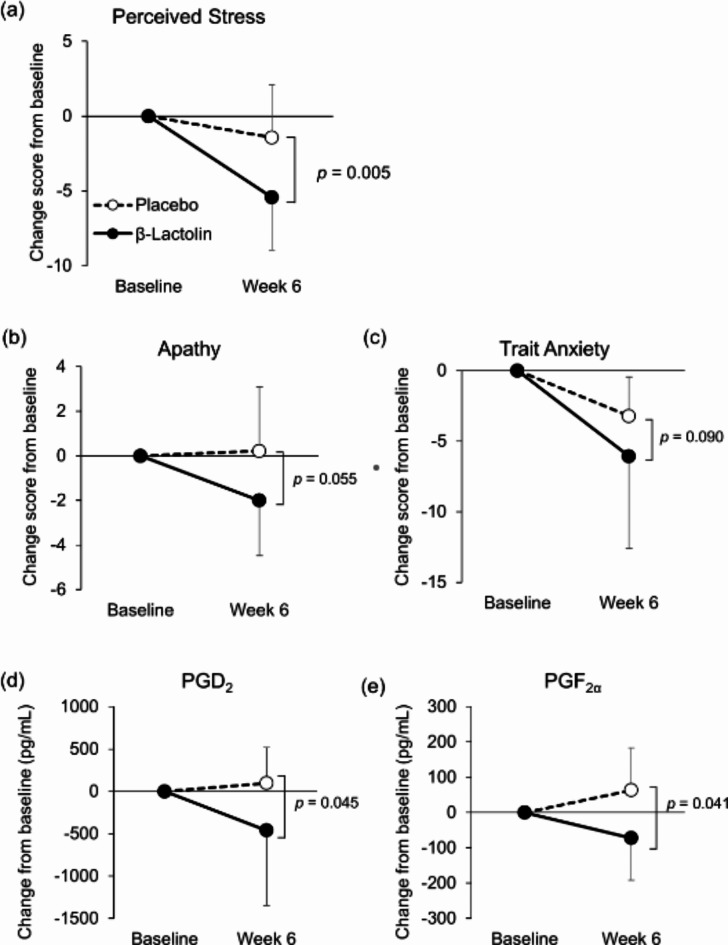
Changes in mood states and salivary PG levels in the 45- to 54-year-old subgroup. (**a**–**c**) Mood states were analyzed in 45–54 years subgroup. Score changes in (**a**) perceived stress, (**b**) apathy scale, and (**c**) trait anxiety from baseline to week 6 were compared between each group. (**d**, **e**) Salivary levels of lipid metabolites were analyzed in the 45–54 years subgroup. Changes in (**d**) PGD_2_ and (**e**) PGF_2α_ levels were compared between each group. The solid line indicates the β-lactolin group (*n* = 14), and the dotted line indicates the placebo group (*n* = 14). Data are presented as means ± SD. The *p* value shows the between-group difference performed using (**a**–**c**) Mann–Whitney *U* tests and (**d**, **e**) unpaired *t* tests.

**Fig. 9:**
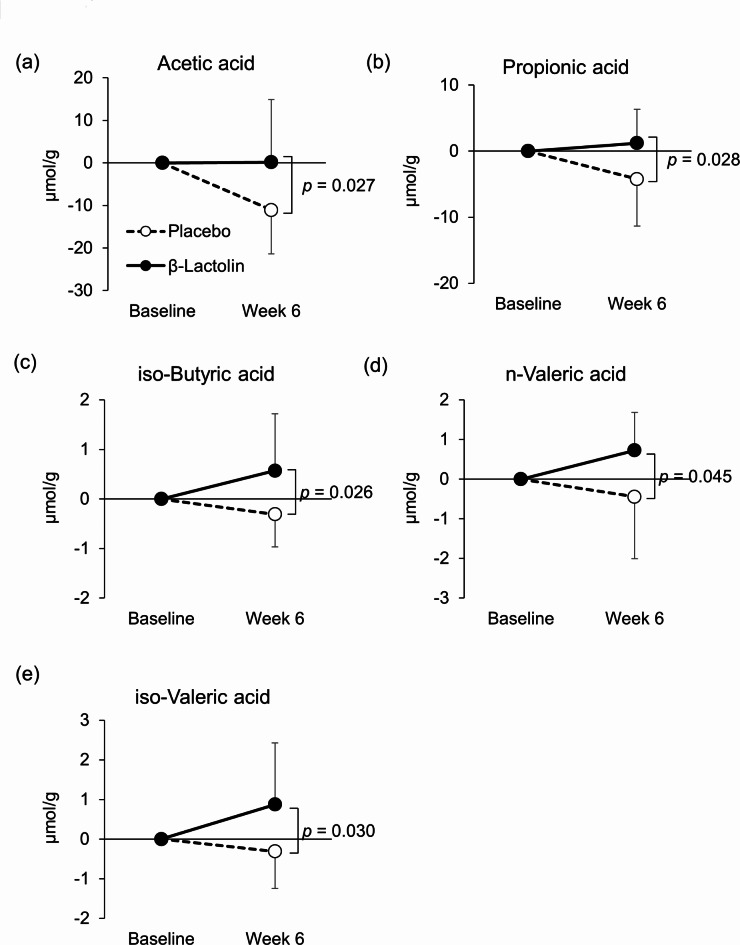
Changes in fecal short-chain fatty acid levels in the 55- to 64-year-old subgroup. Changes in levels in (**a**) acetic acid, (**b**) propionic acid, (**c**) isobutyric acid, (**d**) n-valeric acid, and (**e**) isovaleric acid from baseline to week 6 in the 55–64 years subgroup were compared between each group. The solid line indicates the β-lactolin group (*n* = 14), and the dotted line indicates the placebo group (*n* = 14). Data are presented as means ± SD. The *p* value shows the between-group difference using unpaired *t* tests.

Because the prevalence of mental disorders is affected by age^21^, we performed subgroup analysis based on the age of the subjects. Table 5 presents the characteristics of the subgroups divided by the median age (54.5 years). In the subgroup of subjects aged 45–54 years, changes in PSS score were significantly decreased in the β-lactolin group compared with the placebo group (*p* = 0.005; Fig. 8), whereas this change was not observed in the 55–64 years subgroup (Table 6). Changes in the apathy scale and trait anxiety in the STAI tended to decrease in the β-lactolin group (*p* = 0.055 and *p* = 0.090, respectively; Fig. 8). In addition, changes in the levels of salivary PGD_2_ and PGF_2α_ were significantly decreased in the β-lactolin group compared with the placebo group (*p* = 0.045 and *p* = 0.041, respectively; Fig. 8). In the subgroup of subjects aged 55–64 years, changes in these parameters were the same between the groups. On the other hand, changes in SCFA levels such as acetic acid, propionic acid, isobutyric acid, n-valeric acid, and isovaleric acid were significantly increased in the 55–64 years subgroup (*p* = 0.027, *p* = 0.028, *p* = 0.026, *p* = 0.045, and *p* = 0.030, respectively), although these changes were not observed in the 45–54 years subgroup (Table 7; Fig. 9). Changes in microbiome composition in major phyla and genera were also analyzed in subgroups by age (Supplementary Tables 5, 6). In the subgroup of subjects aged 55–64 years, changes in the *Bacteroides* genus were significantly increased in the β-lactolin group compared with the placebo group (*p* = 0.018, Supplementary Table 6), while changes in the *Bifidobacterium* genus were significantly decreased in the β-lactolin group in the 45–54 years subgroup (*p* = 0.016, Supplementary Table 6).

**Table 5:** Subject characteristics in subgroup analysis by age.

Characteristics	Age < 54.5 (*n* = 28)			54.5 < Age (*n* = 28)		
	Placebo (*n* = 14)	β-lactolin (*n* = 14)	*p* value	Placebo (*n* = 14)	β-lactolin (*n* = 14)	*p* value
Age	49.0 ± 3.1	50.6 ± 3.2	0.193	58.1 ± 2.6	58.2 ± 2.7	0.888
Male/female	9/5	10/4	1.000	11/3	9/5	0.678
Height (cm)	166.26 ± 8.97	167.09 ± 7.89	0.796	168.81 ± 0.71	163.71 ± 6.85	0.075
Body weight (kg)	61.12 ± 9.02	60.30 ± 9.08	0.812	62.29 ± 8.49	60.52 ± 9.19	0.602
Body mass index (kg/m^2^)	22.01 ± 1.55	21.51 ± 2.14	0.485	21.81 ± 2.20	22.48 ± 2.15	0.426
GHQ score	6.1 ± 2.6	6.2 ± 2.9	0.723	6.0 ± 2.6	6.9 ± 3.4	0.541

Data are presented as means ± SD. The *p* value shows between-group difference performed using unpaired *t* tests for age, height, body weight, body mass index, and GHQ test, using χ^2^ test for male/female.

**Table 6:** Scores and score changes for mood state in subgroup analysis by age.

			Age < 54.5 (*n* = 28)			54.5 < Age (*n* = 28)		
			Week 0	Week 6	Changes from baseline	Week 0	Week 6	Changes from baseline
Apathy scale	Placebo	13.9 ± 5.8	14.1 ± 6.6	0.2 ± 2.9		14.1 ± 6.7	13.4 ± 5.3	-0.7 ± 4.9
		β-lactolin	16.1 ± 5.7	14.1 ± 4.7	-2.0 ± 2.5	14.4 ± 3.8	12.6 ± 5.2	-1.8 ± 3.7
		*p* value	0.158	0.708	0.055	0.937	0.865	0.547
BDI-II		Placebo	11.7 ± 4.2	7.5 ± 3.6	-4.1 ± 4.1	11.9 ± 4.5	8.1 ± 5.6	-3.7 ± 4.5
		β-lactolin	11.7 ± 3.7	5.2 ± 4.1	-6.5 ± 4.9	13.0 ± 3.4	7.9 ± 5.2	-5.1 ± 4.1
		*p* value	0.937	0.145	0.248	0.341	0.918	0.344
STAI	State anxiety	Placebo	42.9 ± 7.8	37.9 ± 5.7	-4.9 ± 5.9	42.3 ± 7.6	38.8 ± 7.0	-3.5 ± 5.9
		β-lactolin	46.7 ± 8.1	42.2 ± 4.3	-4.5 ± 5.5	43.2 ± 7.6	38.2 ± 8.1	-5.0 ± 5.2
		*p* value	0.213	0.053	0.991	0.675	0.883	0.577
	Trait anxiety	Placebo	45.9 ± 7.3	42.7 ± 8.2	-3.2 ± 2.8	46.4 ± 7.1	42.3 ± 6.1	-4.1 ± 3.9
		β-lactolin	50.4 ± 7.0	44.3 ± 5.5	-6.1 ± 6.5	44.1 ± 5.4	38.2 ± 8.5	7-5.9 ± 5.2
		*p* value	0.110	0.675	0.090	0.321	0.040	0.276
	Total	Placebo	88.8 ± 13.3	80.6 ± 11.6	-8.1 ± 7.3	88.6 ± 13.6	81.1 ± 12.3	-7.6 ± 9.0
		β-lactolin	97.1 ± 13.0	86.5 ± 8.5	10.6 ± 9.7	87.4 ± 10.4	76.4 ± 4.8	-10.9 ± 9.5
		*p* value	0.060	0.279	0.474	0.777	0.214	0.563
PSS		Placebo	39.0 ± 4.0	37.6 ± 4.5	-1.4 ± 3.5	39.3 ± 6.2	36.5 ± 5.6	-2.8 ± 4.8
		β-lactolin	41.7 ± 4.4	36.3 ± 4.2	-5.4 ± 3.6	37.2 ± 5.4	33.6 ± 5.5	-3.6 ± 5.7
		*p* value	0.104	0.472	0.005	0.345	0.143	0.742
S-H resilience test	Placebo	96.3 ± 14.7	101.6 ± 15.9	5.4 ± 6.6		97.3 ± 13.3	100.9 ± 10.6	3.6 ± 7.4
		β-lactolin	91.9 ± 10.8	100.1 ± 12.5	8.2 ± 5.5	95.9 ± 11.6	99.6 ± 11.7	3.8 ± 7.8
		*p* value	0.312	0.447	0.166	0.760	0.642	0.919
FFMQ	Observing	Placebo	21.4 ± 5.5	21.7 ± 6.0	0.3 ± 2.3	20.5 ± 5.1	20.9 ± 4.9	0.4 ± 2.5
		β-lactolin	23.5 ± 6.4	22.6 ± 7.3	-0.9 ± 1.5	20.3 ± 4.9	20.1 ± 4.1	-0.2 ± 3.4
		*p* value	0.578	0.848	0.09	0.973	0.991	0.624
	Nonreactivity	Placebo	20.7 ± 2.6	21.3 ± 2.7	0.6 ± 2.3	20.4 ± 4.1	22.6 ± 3.3	2.1 ± 2.6
		β-lactolin	19.2 ± 3.0	20.4 ± 2.6	1.1 ± 2.0	20.9 ± 3.5	22.4 ± 4.8	1.6 ± 4.2
		*p* value	0.193	0.469	0.512	0.829	0.937	0.307
	Nonjudging	Placebo	26.9 ± 4.2	26.5 ± 3.6	-0.4 ± 3.4	27.5 ± 3.3	28.0 ± 3.2	0.5 ± 2.4
		β-lactolin	25.6 ± 3.1	27.5 ± 5.1	1.9 ± 4.0	28.8 ± 3.7	29.3 ± 3.6	0.5 ± 2.6
		*p* value	0.417	0.725	0.248	0.332	0.380	0.738
	Describing	Placebo	24.4 ± 5.5	24.9 ± 6.2	0.5 ± 3.2	23.7 ± 5.6	25.5 ± 5.7	1.8 ± 2.8
		β-lactolin	22.8 ± 5.2	24.2 ± 5.3	1.4 ± 2.4	25.0 ± 6.2	25.0 ± 5.4	0.0 ± 1.8
		*p* value	0.394	0.829	0.592	0.760	0.847	0.033
	acting with Awareness	Placebo	26.7 ± 3.7	27.6 ± 4.6	0.9 ± 2.8	26.7 ± 3.8	27.8 ± 3.7	1.1 ± 3.3
		β-lactolin	25.6 ± 4.1	27.1 ± 4.4	1.6 ± 3.8	28.6 ± 3.7	29.8 ± 3.8	1.2:2.1
		*p* value	0.380	0.691	0.590	0.098	0.150	0.919

Data are presented as means ± SD. The *p* value shows the between-group difference performed using Mann–Whitney *U* tests. β-lactolin group (*n* = 14, in both subgroup); placebo group (*n* = 14, in both subgroup); *p < 0.05, **p < 0.01 compared with baseline in each group using paired *t* tests; #*p* < 0.05 in Mann–Whitney *U* tests. BDI–II, Beck Depression Inventory–Second Edition; STAI, State–Trait Anxiety Inventory; PSS, Perceived Stress Scale; FFMQ, Five Facet Mindfulness Questionnaire.

**Table 7:** Fecal short-chain fatty acid levels in subgroup analysis by age.

		Age < 54.5 (*n* = 28)			54.5 < Age (*n* = 28)		
		Week 0	Week 6	Changes from baseline	Week 0	Week 6	Changes from baseline
Acetic acid	Placebo	38.537 ± 15.890	36.742 ± 13.530	-1.796 ± 24.741	42.890 ± 13.433	31.806 ± 10.666	-11.083 ± 10.300
	β-lactolin	36.908 ± 17.091	33.055 ± 13.780	-3.852 ± 19.662	36.518 ± 16.095	36.698 ± 11.098	0.180 ± 14.738
	*p* value	0.796	0.481	0.810	0.266	0.245	0.027
Propionic acid	Placebo	13.123 ± 9.502	13.743 ± 5.063	0.350 ± 9.080	14.708 ± 8.956	10.479 ± 5.017	-4.229 ± 7.119
	β-lactolin	12.462 ± 7.727	11.966 ± 7.090	-0.496 ± 6.018	10.880 ± 5.332	12.097 ± 5.463	1.217 ± 5.137
	*p* value	0.842	0.523	0.774	0.181	0.422	0.028
n-Butyric acid	Placebo	7.241 ± 3.374	8.367 ± 5.532	1.126 ± 5.987	10.388 ± 6.259	8.521 ± 8.798	-1.866 ± 9.160
	β-lactolin	9.864 ± 6.291	8.851 ± 6.687	-1.013 ± 6.116	7.664 ± 4.276	10.448 ± 5.422	2.784 ± 4.077
	*p* value	0.181	0.836	0.358	0.190	0.492	0.094
iso-Butyric acid	Placebo	1.298 ± 0.619	1.557 ± 0.679	0.258 ± 1.058	1.659 ± 1.028	1.350 ± 0.567	-0.309 ± 0.654
	β-lactolin	1.534 ± 0.896	1.414 ± 1.557	-0.120 ± 0.976	1.247 ± 0.621	1.820 ± 1.090	0.573 ± 1.147
	*p* value	0.465	0.677	0.363	0.242	0.185	0.026
n-Valeric acid	Placebo	1.797 ± 0.886	2.110 ± 1.159	0.313 ± 1.351	2.137 ± 1.705	1.694 ± 0.771	-0.442 ± 1.563
	β-lactolin	1.898 ± 1.279	1.738 ± 1.106	-0.159 ± 1.281	1.470 ± 0.842	2.196 ± 1.138	0.726 ± 0.959
	*p* value	0.834	0.443	0.401	0.255	0.226	0.045
iso-Valeric acid	Placebo	1.926 ± 1.098	2.379 ± 1.134	0.453 ± 1.815	2.420 ± 1.518	2.111 ± 0.906	-0.309 ± 0.940
	β-lactolin	2.510 ± 1.423	2.204 ± 1.493	-0.306 ± 1.591	1.850 ± 1.110	2.723 ± 1.613	0.873 ± 1.555
	*p* value	0.279	0.754	0.287	0.299	0.249	0.030
n-Capronic acid	Placebo	0.824 ± 0.854	0.687 ± 0.365	-0.136 ± 0.974	0.523 ± 0.139	0.481 ± 0.123	-0.042 ± 0.188
	β-lactolin	1.092 ± 0.702	0.453 ± 0.252	-0.639 ± 0.838	0.726 ± 0.400	1.022 ± 0.909	0.296 ± 0.623
	*p* value	0.630	0.312	0.441	0.320	0.222	0.281

Data are presented as means ± SD. The *p* value shows the between-group difference performed using unpaired *t* tests. β-lactolin group (*n* = 14); placebo group (*n* = 14); *p < 0.05 compared with baseline in each group using paired *t* tests; #*p* < 0.05 in unpaired *t* tests.

### Assessment of safety

To evaluate the safety of the intervention, clinical examinations and medical interviews were performed by the principal investigator. No clinical values were changed from the baseline. Ten subjects in the placebo group and 15 subjects in the β-lactolin reported adverse events during the study, but none of these were related to the interventions.

## Discussion

In this randomized double-blind placebo-controlled study, we evaluated the effects of β-lactolin supplementation on mood states in healthy middle- and older-aged adults. We found that trait anxiety evaluated by the STAI and perceived stress evaluated by the PSS were improved after supplementation with β-lactolin for 6 weeks. Although we did not observe statistically significant differences, the β-lactolin–supplemented group showed better changes in other aspects of mood state, such as apathy state evaluated by the apathy scale and depressive mood evaluated by BDI–II. These results suggest that the daily intake of β-lactolin improves various aspects of mood states and especially improves trait anxiety and subjective stress. In addition, in the assessment of health-related QOL by the SF-36, the vitality subscale and the psychological summary score were significantly improved in the β-lactolin–supplemented group. Improvement in trait anxiety and subjective stress by β-lactolin supplementation may lead to an improvement in psychological health-related QOL.

To confirm the effect of β-lactolin on anxiety and subjective stress improvement using biochemical measurements, we evaluated changes in salivary stress markers. Although there were no significant differences in cortisol and α-amylase levels, daily intake of β-lactolin kept salivary IgA levels higher than placebo intake did. Salivary IgA has been regarded as a marker of immunological stress, which is decreased in the chronic stress condition^22^. An increase in salivary IgA level by β-lactolin supplementation would be related to the improvement in anxiety and subjective stress. This finding led us to perform a post hoc analysis of LM salivary levels related to immunological responses, although we observed no significant differences. It has recently been reported that IgA levels interact with the gut microbiome^23^. In the present study, the composition of the *Actinobacteria* phylum and *Bifidobacterium*genus changed after β-lactolin treatment, although it is difficult to interpret these changes because of the differences in composition between the groups at baseline. We have not elucidated whether β-lactolin reach the colon directly, but it is well known that tryptophan is metabolized by gut microbiota, so at least some fragments of this peptide could affect the gut microbiome. And also, it has been reported that the induction of IgA by gut bacteria is different between strains^24,25^. The effect on the gut microbiome or immunological function may underlie the mechanism of β-lactolin; however, to elucidate these points, further studies focusing on gut microbiome are needed.

In the present study, we explored the relationship between baseline mood states and salivary LMs. We found that the levels of PGD_2_ and its metabolite 15d-PGJ_2_ were positively correlated with anxiety and perceived stress. Previously, researchers reported that salivary levels of PGD_2_, PGE_2_, and PGF_2α_were significantly higher in patients with depression^26^. Thus, the results of the present study, in which subjects with worse anxiety or subjective stress were found to exhibit higher PGs, may be consistent with the findings of previous studies. In addition, we discovered for the first time that salivary linoleic acid metabolites such as HYB and Keto B were positively correlated with anxiety and perceived stress. It is known that linoleic acids are metabolized by intestinal bacteria to produce linoleic acid metabolites, which have been reported to exhibit various physiological functions including an immunomodulatory effect^27,28^and improvement in energy metabolism^29,30^. However, no studies to date have reported a relationship with brain functions, including mood states. Ours is the first report to measure these compounds in human salivary samples. Although these LMs showed a relatively low correlation, these findings might lead to the establishment of a novel, easy, and noninvasive method of evaluating human anxiety and subjective stress, which would be beneficial for screening preclinical mental disturbances.

Researchers have reported that the prevalence of mental disorders is highest among those of middle age (e.g., 45–54 years old)^21^. We performed a subgroup analysis based on the median subject age (54.5 years old) and found that the subjective stress in the younger subgroup was greatly improved by β-lactolin supplementation (*p* = 0.005, Fig. 8). We also noted that score changes on the apathy scale and trait anxiety also tended to improve in the β-lactolin group. β-Lactolin seems to improve subjective stress in those aged 45–54 years, whereas other scores on the mood state assessments were not significantly changed. We hypothesized that the baseline mood states were lower in the 45–54 years subgroup compared with those in the 55–64 years subgroup, although there was no difference between subgroups in baseline scores of the mood state assessments. Additional studies should further examine the reason why β-lactolin markedly improved subjective stress. Interestingly, salivary PGD_2_ and PGF_2α_ levels were significantly reduced by β-lactolin supplementation in the 45–54 years subgroup (Fig. 8). β-lactolin has been reported to suppress the inflammatory responses in aged rodents^31^; thus, the anti-inflammatory function of β-lactolin may underlie the improvement of perceived stress in the 45–54 years subgroup. On the other hand, in the subgroup of those aged 55–64 years, fecal SCFAs were significantly increased in the β-lactolin group, although there were no intergroup differences in mood state changes. Changes in the *Bacteroides* genus were significantly increased in the β-lactolin group in the 55–64 years subgroup. It is generally known that *Bacteroides* genus produces SCFAs, so the increase in *Bacteroides*abundance by β-lactolin may be involved in the SCFA increase. Aging is known to affect the gut microbiome^32^; thus, the effects of β-lactolin on the gut environment may vary between ages. Again, future studies should investigate age-related differences in the effect of β-lactolin.

Previous preclinical studies have shown that β-lactolin improves memory function through the increase of dopamine release and the activation of the dopamine D1 receptor, and these functions may be mediated by the inhibition of monoamine oxidase-B^12,14^. Another preclinical study showed that the administration of β-lactolin suppressed depressive-like behavior, which was diminished by treatment with dopamine receptor inhibitor in the rodent behavioral tests^15^. Although we did not examine the involvement of the dopaminergic system in this clinical trial, β-lactolin may improve anxiety and subjective stress via dopamine modulation. It has been reported that the breakdown of the dopaminergic system is associated with various psychological disorders or mood state imbalance, such as major depression^33^and anxiety disorders^34^. The modulation of dopaminergic systems in daily life may be effective for preventing these mental disturbances. On the other hand, the relationship between the dopaminergic system and trait anxiety have not been clearly investigated to date; thus, other mechanisms such as the gut–brain interaction or immunological pathway may underlie the effect of β-lactolin. We have previously demonstrated that supplementation with β-lactolin enhances CBF in the dorsolateral prefrontal cortex as assessed in near-infrared spectroscopy during cognitive tasks^18,19^. Associations between dorsolateral prefrontal CBF and anxiety have been reported^35,36^; therefore, CBF increase by β-lactolin may underlie the improvement in anxiety and subjective stress.

This study has several limitations. First, primary outcomes were measured using subjective scales, and learning effects were therefore not completely eliminated. Second, mechanisms related to the dopaminergic system or CBF must be evaluated in a future study. It is necessary to evaluate mood state–related brain function combined with objective measurements of CBF or imaging dopaminergic function in future studies.

In conclusion, in the present study, we revealed for the first time that daily supplementation with β-lactolin–rich whey peptides improves trait anxiety and subjective stress. Mechanistically, β-lactolin may affect the levels of immunological mediators, which were detected in subject saliva measurements. The serial results propose an effective strategy for preventing a decline in mood state and onset of mood disorders through the establishment of daily dietary habits.

### Methods

### Subjects

We recruited 247 Japanese-speaking healthy adults aged 45–64 years who reported often feeling annoyed or anxiety in daily life. Subjects with relatively low scores in the GHQ-28 were preferentially included. We also included subjects with a score of < 19 on the BDI–II. The exclusion criteria were (1) habitual intake of drugs, supplements, or foods rich in whey peptide (2); habitual intake of supplements or foods rich in protein (3); habitual intake of drugs or foods that may affect mood, fatigue, stress, and sleep (4); regular activities to improve mood, fatigue, stress, and sleep (5); irregular working days (6); irregular lifestyles (7); plans for a great change in lifestyle (8); high habitual consumption of alcohol (> 20 g/day) (9); diagnosed psychological disorders (10); diagnosed sleep disorders (11); diagnosed chronic fatigue syndrome (12); allergy to milk (13); lactose intolerance (14); intake of antibiotics within 3 months before fecal sampling (15); unsuitable feces for fecal sampling (16); smoking habit (17); diagnosed dry mouth (18); treatment for tooth or mouth within 1 month (19); unusual state in mouth (20); bleeding in the mouth when brushing teeth at least once per week (21); blood transfusion or blood donation within 3 months (22); participation in any other clinical studies within 1 month (23); present or past history of serious disease (24); pregnant or breastfeeding.

In previous studies of other functional food (i.e., a theanine-rich matcha green tea), 39 subjects were required in order to detect a significant differences in anxiety as assessed using the STAI^37^. With an assumption of a 10% withdrawal rate, at least 44 subjects were required in this study.

### Test supplements

Test tablets containing 1.6 mg of β-lactolin in 1 g of whey peptide were prepared, and placebo tablets consisted of a substitution of 1 g of maltodextrin. The same composition has been used for previous clinical trials, and the safety has been confirmed^16,17^. The test supplements were the same shape and size as those of the test tablets and indistinguishable by taste. Tablets were ingested by each group subjects every day for 6 weeks.

### Procedures

We designed this study as a randomized, placebo-controlled, double-blind, parallel-group comparative study. In the first screening step, we prepared questionnaires for inclusion and exclusion criteria and lifestyle data, measurements of blood pressure, pulse rate, height and body weight, GHQ-28 scores, and assessment of mood states. Because the GHQ-28 is a widely used screening test for mental disorders, we used it to screen subjects with lower mood states. Blood and urine collection, clinical examinations, and medical interview by the principal investigator for assessments of safety were also performed in the first screening step. Subjects who met the inclusion criteria and did not meet the exclusion criteria proceeded to the second screening step. In the second screening step, we conducted a medical interview, measured blood pressure and pulse rate, collected saliva, and assessed mood states. Subjects who fulfilled the eligibility criteria were allocated in a 1:1 ratio to the β-lactolin or placebo groups by a computer-programmed minimization method that ensured balanced age, sex, BDI–II scores, and STAI scores between each group. The allocator was not involved in determining subject eligibility, data collection, or analysis. Subjects, research staff, and outcome assessors were blinded to group allocations until data analyses were completed. We assessed mood states and collected saliva in the second screening step (baseline measurement) and after 6 weeks of intervention. The SF-36 was administered after 0, 2, 4, and 6 weeks of intervention. Subjects answered the questionnaire regarding stress events every week after the second screening step until the end of the intervention period. A diet survey followed by fecal sampling was conducted after 0 and 6 weeks of intervention. Clinical examinations and medical interview for assessment of safety were performed after 6 weeks of intervention. Subjects were instructed to maintain their regular lifestyles, including diet, exercise, and sleep habits, and to avoid taking any drugs, health foods, or protein supplements that could affect mood states during the study. Subject diaries were used to monitor compliance with instructions. On the day of the mood state assessments, subjects were instructed to completely avoid consuming any foods or beverages except for water for 2 h before the start of the mood state assessments. All assessments were performed at Medical Station Clinic (Tokyo, Japan) between August 2019 and February 2020. All assessments were completed before the outbreak of COVID-19 in Japan.

### Mood state assessments (primary outcomes)

To evaluate the multiple aspects of mood states, we administered the following questionnaires. Depressive mood states were evaluated using the BDI–II^38^. Anxiety was evaluated using the STAI^39^. The STAI was composed of a state anxiety assessment and trait anxiety assessment. In this study, we compared the scores of state anxiety and trait anxiety and the total score between groups. Subjective stress was evaluated using the PSS^40^. Resilience was evaluated using the S-H resilience test^41,42^. Mindfulness, a state with awareness of the present moment in a nonreactive and nonjudgmental manner, was evaluated using the FFMQ^43^. The FFMQ had five components (Observing, Nonreactivity, Nonjudging, Describing, and acting with Awareness), which we analyzed individually.

### Health-related QOL assessments

Health-related QOL was assessed using the SF-36. The SF-36 was composed of eight subscales: physical functioning (PF), role limitations due to physical health (PR), bodily pain (BP), general health perceptions (GH), vitality (VT), social functioning (SF), role limitations due to emotional problems (RE), and mental health (MH). Summary scores adjusted to Japanese subjects, physical component scores (PCS) and mental component scores (MCS), were also calculated. Subjects were instructed to answer the SF-36 questionnaire at home after 0, 2, 4, and 6 weeks of intervention.

### Mental stress markers in saliva

To evaluate the levels of biochemical stress markers, saliva samples were collected for 2 min using Salisoft (Sarstedt, Germany), centrifuged at 2,000 rpm for 5 min at room temperature. The supernatants were collected to microtubes and immediately frozen until measurements. Salivary cortisol, α-amylase, and IgA were measured using a salivary cortisol EIA kit (Salimetrics, State College, PA), salivary α-amylase kinetic enzyme assay kit (Salimetrics), and salivary secretory IgA EIA kit (Salimetrics), respectively.

### Fecal short-chain fatty acids and microbiome analysis

Fecal samples were collected at weeks 0 and 6 of the intervention by each participant into a commercially available feces collection kit (TechnoSuruga Laboratory Co. Ltd, Shizuoka, Japan). Collected samples were immediately refrigerated and sent to TechnoSuruga Laboratory Co. Ltd. (Shizuoka, Japan) and subjected toSCFA determination and DNA sequencing. The amount of SCFAs was determined using gas chromatography (GC) as previously described. Briefly, 100 mg of each sample was suspended with 900 mg of 0.5% (v/v) phosphoric acid and heated at 85 °C for 15 min. Samples were centrifuged at 14,000 rpm for 10 min and supernatants subsequently collected. An equal volume of ethyl acetate was added and then centrifuged again at 14,000 rpm for 10 min. The ethyl acetate layer was collected and added to 4-methylvaleric acid for internal standard, which was then subjected to GC measurement.

We analyzed the fecal microbiome with 16 S rDNA of the prokaryotes, following the previously described procedures^44^. Briefly, 100 mg of each sample was suspended in 4 M guanidium thiocyanate, 100 mM Tris-HCl (pH 9.0), and 40 mM EDTA. DNA samples were extracted using an automatic DNA separator (GENE PREP STAR PI-480; Kurabo Industries, Osaka, Japan) and reagent kit (NR-201; Kurabo Industries). After the DNA concentrations were determined, the 16 S rDNA sequences were amplified by PCR, as previously described^44^. The sequences were determined by the next-generation sequence amplicon analysis using the Illumina MiSeq platform (Illumina, San Diego, CA) and MiSeq Reagent Kit v3 (Illumina). We identified the bacteria using the TechnoSuruga Lab Microbial Identification database DB-BA 13.0 (TechnoSuruga Laboratory) and Metagenome@KIN software (World Fusion Co., Tokyo, Japan) at a 97% sequence similarity. Relative abundances were calculated as the ratio of the read counts of specific bacterium to the total 16 S rDNA.

### Salivary lipid metabolites (post hoc analysis)

After the primary and secondary outcomes were fixed, we further investigated the resting salivary samples. LM lipidomics analysis was performed as described previously^45^. We incubated 100 µL of saliva for 1 h at 4 °C with 1 mL of methanol for total lipid extraction. Deuterium (d)–labeled internal standards, d8-5 S-hydroxy-eicosatetraenoic acid (5-HETE), d4-leukotriene (LT) B_4_, d4- PGE_2_, and d5-resolvin (Rv) D2 were added to facilitate the quantification of sample recovery. All samples for lipidomics were extracted using C18 solid-phase extraction columns and subjected to liquid chromatography–tandem mass spectrometry (LC–MS/MS). The LC–MS/MS system, QTrap 6500 (Sciex, Boston, MA, USA), was equipped with a Nexera X2 HPLC system (Shimadzu Corporation, Kyoto, Japan). We used an Agilent Eclipse Plus C18 column (100 mm × 4.6 mm × 1.8 μm) with a gradient of methanol: water: acetic acid of 55:45:0.01 (v: v:v) to 98:2:0.01 at a 0.4 mL/min flow rate. To monitor and quantify the levels of targeted LM, a multiple-reaction monitoring (MRM) method was devised with signature ion fragments for each molecule. Identification was conducted using published criteria^45^ using LC retention times, specific fragmentation patterns, and at least six diagnostic fragmentation ions. Quantification was performed based on the peak area of the MRM transition, and linear calibration curves were obtained using an authentic standard for each compound. Experimenters under blinded condition measured the LMs.

### Statistical analysis

Research staff under blinded condition performed the data analysis according to a predefined plan. All results were expressed as means ± standard value. Statistical analysis was performed using IBM SPSS Statistics 26 (IBM, New York, NY). For comparisons of primary outcomes, the change of scores between baseline and week 6 were compared between groups using Mann–Whitney *U* tests. The change from baseline to week 6 within each group was also compared using Wilcoxon signed-rank tests. The change in the SF-36 scores from baseline to weeks 2, 4, and 6 were compared between groups using unpaired *t* tests. For all other results, the change in scores from baseline to week 6 were compared between groups using unpaired *t* tests. Subgroup analysis based on the age of the subjects, which divided by the median age (54.5 years), was performed. The correlation between baseline mood state scores and salivary LM levels were examined using Spearman correlation coefficient.

## Electronic supplementary material

Below is the link to the electronic supplementary material.

Supplementary Material 1

## Data Availability

Data described in the manuscript, code book, and analytic code will be made available from the corresponding author upon reasonable request.
