# Supplementary material for: Supplementation with whey peptide rich in β-lactolin improves trait anxiety and subjective stress in healthy adults: a randomized, double-blind, placebo-controlled study
Source: Sci Rep. 2024 Oct 8;14:23444. doi: 10.1038/s41598-024-73780-3 (PMC11461648; doi:10.1038/s41598-024-73780-3)
Supplement: Supplementary file 1 — Supplementary Material 1 [file 41598_2024_73780_MOESM1_ESM.docx]

**Supplementary Table 1. Changes in short chain fatty acid levels.**

|  |  | Baseline (Week 0) | Week 6 | Changes from baseline |
| --- | --- | --- | --- | --- |
| Acetic acid | Placebo | 40.714 ± 14.607 | 34.274 ± 12.216 | -6.440 ± 19.188 |
|  | β-lactolin | 36.713 ± 16.291 | 34.877 ± 12.417 | -1.836 ± 17.174 |
|  | p value | 0.338 | 0.855 | 0.348 |
| Propionic acid | Placebo | 13.915 ± 9.096 | 11.976 ± 5.176 | -1.939 ± 8.339 |
|  | β-lactolin | 11.671 ± 6.564 | 12.031 ± 6.211 | 0.360 ± 5.559 |
|  | p value | 0.294 | 0.971 | 0.23 |
| n-Butyric acid | Placebo | 8.814 ± 5.188 | 8.444 ± 7.212 | -0.370 ± 7.745 |
|  | β-lactolin | 8.764 ± 5.396 | 9.650 ± 6.029 | 0.886 ± 5.454 |
|  | p value | 0.972 | 0.5 | 0.486 |
| iso-Butyric acid | Placebo | 1.494 ± 0.867 | 1.445 ± 0.616 | -0.049 ± 0.891 |
|  | β-lactolin | 1.402 ± 0.780 | 1.601 ± 1.017 | 0.199 ± 1.094 |
|  | p value | 0.694 | 0.519 | 0.386 |
| n-Valeric acid | Placebo | 1.982 ± 1.374 | 1.883 ± 0.965 | -0.099 ± 1.487 |
|  | β-lactolin | 1.702 ± 1.100 | 1.948 ± 1.121 | 0.246 ± 1.208 |
|  | p value | 0.447 | 0.835 | 0.39 |
| iso-Valeric acid | Placebo | 2.193 ± 1.338 | 2.234 ± 1.003 | 0.040 ± 1.429 |
|  | β-lactolin | 2.193 ± 1.300 | 2.453 ± 1.542 | 0.260 ± 1.654 |
|  | p value | 1 | 0.559 | 0.622 |
| n-Capronic acid | Placebo | 0.674 ± 0.598 | 0.584 ± 0.279 | -0.089 ± 0.663 |
|  | β-lactolin | 0.909 ± 0.564 | 0.737 ± 0.688 | -0.171 ± 0.847 |
|  | p value | 0.408 | 0.529 | 0.82 |

Data are presented as means ± SD. The *p* value shows the between-group difference using unpaired *t* tests.

**Supplementary Table 2. Changes in microbiome phylum composition**

|  |  | Baseline (Week 0) | Week 6 | Changes from baseline |
| --- | --- | --- | --- | --- |
| Firmicutes | Placebo | 60.68 ± 12.14 | 61.23 ± 14.20 | 0.55 ± 10.03 |
|  | β-lactolin | 52.78 ± 11.85# | 56.57 ± 14.48 | 3.79 ± 11.89 |
|  | p value | 0.017 | 0.229 | 0.275 |
| Actinobacteria | Placebo | 21.10 ± 12.67 | 20.50 ± 12.57 | -0.61 ± 11.89 |
|  | β-lactolin | 27.23 ± 14.86 | 19.96 ± 13.19** | -7.27 ± 7.76# |
|  | p value | 0.103 | 0.877 | 0.016 |
| Bacteroidetes | Placebo | 4.69 ± 5.11 | 5.55 ± 4.54 | 0.86 ± 5.50 |
|  | β-lactolin | 5.61 ± 5.90 | 8.26 ± 6.82* | 2.65 ± 6.05 |
|  | p value | 0.537 | 0.085 | 0.25 |
| Proteobacteria | Placebo | 2.29 ± 5.39 | 0.96 ± 1.80 | -1.33 ± 3.97 |
|  | β-lactolin | 1.05 ± 2.89 | 1.33 ± 3.41 | 0.28 ± 2.48 |
|  | p value | 0.286 | 0.617 | 0.074 |
| Verrucomicrobia | Placebo | 0.19 ± 0.40 | 0.26 ± 0.79 | 0.07 ± 0.71 |
|  | β-lactolin | 0.33 ± 1.32 | 0.12 ± 0.29 | -0.21 ± 1.31 |
|  | p value | 0.57 | 0.396 | 0.315 |

Data are presented as means ± SD. β-lactolin group (n = 28); placebo group (n = 28); **p* < 0.05, ***p* < 0.01 compared with baseline in each group using paired *t* tests; #*p* < 0.05 in unpaired *t* tests.

**Supplementary Table 3. Changes in microbiome genus composition**

|  |  | Baseline (Week 0) | Week 6 | Changes from baseline |
| --- | --- | --- | --- | --- |
| Bifidobacterium | Placebo | 11.34 ± 9.13 | 12.08 ± 9.00 | 0.74 ± 9.58 |
|  | β-lactolin | 18.38 ± 13.93# | 12.32 ± 12.19** | -6.07 ± 7.76## |
|  | p value | 0.029 | 0.934 | 0.005 |
| Bacteroides | Placebo | 2.96 ± 3.17 | 3.75 ± 4.00 | 0.79 ± 4.21 |
|  | β-lactolin | 3.37 ± 4.04 | 5.95 ± 5.83* | 2.58 ± 5.20 |
|  | p value | 0.671 | 0.106 | 0.164 |
| Romboutsia | Placebo | 0.80 ± 1.67 | 0.69 ± 1.04 | -0.11 ± 1.72 |
|  | β-lactolin | 0.92 ± 1.04 | 0.84 ± 1.38 | -0.08 ± 1.28 |
|  | p value | 0.756 | 0.645 | 0.929 |
| Clostridium | Placebo | 0.47 ± 1.55 | 1.18 ± 2.86 | 0.71 ± 2.33 |
|  | β-lactolin | 0.30 ± 0.51 | 0.21 ± 0.42 | -0.09 ± 0.66 |
|  | p value | 0.582 | 0.081 | 0.087 |
| Lactobacillus | Placebo | 0.28 ± 0.75 | 0.89 ± 2.51 | 0.61 ± 1.83 |
|  | β-lactolin | 0.35 ± 0.84 | 0.34 ± 0.62 | -0.01 ± 0.57 |
|  | p value | 0.745 | 0.269 | 0.096 |
| Lactococcus | Placebo | 0.04 ± 0.11 | 0.03 ± 0.10 | -0.01 ± 0.15 |
|  | β-lactolin | 0.01 ± 0.02 | 0.01 ± 0.03 | 0.00 ± 0.04 |
|  | p value | 0.181 | 0.332 | 0.769 |

Data are presented as means ± SD. β-lactolin group (n = 28); placebo group (n = 28); **p* < 0.05, ***p* < 0.01 compared with baseline in each group using paired *t* tests; #*p* < 0.05 in analysis of covariance.

**Supplementary Table 4. Changes in salivary lipid mediator levels**

|  |  |  | Baseline (Week 0) | Week 6 | Changes from baseline | *p* value |
| --- | --- | --- | --- | --- | --- | --- |
| AA | PGE2 | Placebo | 516.130 | 461.241 | -54.888 |  |
|  |  | β-lactolin | 573.030 | 452.994 | -120.037 | 0.427 |
|  | PGD2 | Placebo | 1478.018 | 1194.580 | -283.439 |  |
|  |  | β-lactolin | 1328.427 | 1208.230 | -120.197 | 0.688 |
|  | PGJ2+delta12-PGJ2 | Placebo | 0.000 | 0.000 | 0.000 |  |
|  |  | β-lactolin | 0.000 | 0.000 | 0.000 |  |
|  | 15deoxy-d12,14 PGJ2 | Placebo | 49654.088 | 46030.424 | -3623.664 |  |
|  |  | β-lactolin | 53937.346 | 45028.295 | -8909.051 | 0.553 |
|  | PGF2a | Placebo | 241.893 | 258.655 | 16.762 |  |
|  |  | β-lactolin | 241.855 | 208.436 | -33.419 | 0.193 |
|  | TxB2 | Placebo | 2039.251 | 1792.211 | -247.040 |  |
|  |  | β-lactolin | 2154.892 | 1911.415 | -243.476 | 0.988 |
|  | 12S-HHT | Placebo | 2.968 | 2.559 | -0.409 |  |
|  |  | β-lactolin | 3.461 | 2.440 | -1.021 | 0.535 |
|  | LTB4 | Placebo | 26.737 | 33.674 | 6.937 |  |
|  |  | β-lactolin | 25.571 | 29.289 | 3.718 | 0.476 |
|  | Lipoxin A4 | Placebo | 93.811 | 78.938 | -14.873 |  |
|  |  | β-lactolin | 98.738 | 105.783 | 7.045 | 0.358 |
|  | Lipoxin B4 | Placebo | 0.000 | 0.000 | 0.000 |  |
|  |  | β-lactolin | 0.000 | 0.000 | 0.000 |  |
|  | 5,15-diHETE | Placebo | 28.857 | 27.842 | -1.015 |  |
|  |  | β-lactolin | 27.065 | 31.671 | 4.606 | 0.261 |
| EPA | Lipoxin A5 | Placebo | 0.000 | 0.000 | 0.000 |  |
|  |  | β-lactolin | 0.000 | 0.000 | 0.000 |  |
|  | Resolvin E1 | Placebo | 72.951 | 80.199 | 7.248 |  |
|  |  | β-lactolin | 62.551 | 73.031 | 10.479 | 0.870 |
|  | Resolvin E2 | Placebo | 0.000 | 0.000 | 0.000 |  |
|  |  | β-lactolin | 0.000 | 0.000 | 0.000 |  |
|  | Resolvin E3 | Placebo | 22.804 | 19.880 | -2.924 |  |
|  |  | β-lactolin | 23.628 | 22.008 | -1.619 | 0.691 |
| DHA | Resolvin D1 | Placebo | 0.000 | 0.000 | 0.000 |  |
|  |  | β-lactolin | 0.000 | 0.000 | 0.000 |  |
|  | Resolvin D2 | Placebo | 0.000 | 0.000 | 0.000 |  |
|  |  | β-lactolin | 0.000 | 0.000 | 0.000 |  |
|  | Resolvin D3 | Placebo | 0.000 | 0.000 | 0.000 |  |
|  |  | β-lactolin | 0.000 | 0.000 | 0.000 |  |
|  | Resolvin D5 | Placebo | 0.000 | 0.000 | 0.000 |  |
|  |  | β-lactolin | 0.000 | 0.000 | 0.000 |  |
|  | Protectin D1 | Placebo | 12.685 | 11.958 | -0.727 |  |
|  |  | β-lactolin | 11.360 | 10.373 | -0.988 | 0.861 |
|  | Maresin 1 | Placebo | 0.000 | 0.000 | 0.000 |  |
|  |  | β-lactolin | 0.000 | 0.000 | 0.000 |  |
|  | Maresin 2 | Placebo | 0.000 | 0.000 | 0.000 |  |
|  |  | β-lactolin | 0.000 | 0.000 | 0.000 |  |
| AA | 5-HETE | Placebo | 77.124 | 49.621 | -27.503 |  |
|  |  | β-lactolin | 50.402 | 59.551 | 9.148 | 0.346 |
|  | 12-HETE | Placebo | 2095.032 | 2876.321 | 781.289 |  |
|  |  | β-lactolin | 3579.879 | 3397.788 | -182.091 | 0.366 |
|  | 15-HETE | Placebo | 129.163 | 89.937 | -39.226 |  |
|  |  | β-lactolin | 149.530 | 118.005 | -31.525 | 0.920 |
|  | AA | Placebo | 3005.552 | 2221.107 | -784.445 |  |
|  |  | β-lactolin | 2745.886 | 3119.841 | 373.955 | 0.404 |
| EPA | 5-HEPE | Placebo | 12.880 | 12.179 | -0.701 |  |
|  |  | β-lactolin | 12.506 | 14.071 | 1.565 | 0.376 |
|  | 12-HEPE | Placebo | 206.607 | 267.704 | 61.097 |  |
|  |  | β-lactolin | 274.516 | 249.662 | -24.854 | 0.222 |
|  | 15-HEPE | Placebo | 13.155 | 10.197 | -2.958 |  |
|  |  | β-lactolin | 13.068 | 14.081 | 1.012 | 0.307 |
|  | 18-HEPE | Placebo | 10.750 | 9.783 | -0.967 |  |
|  |  | β-lactolin | 12.764 | 11.251 | -1.513 | 0.853 |
|  | EPA | Placebo | 364.854 | 285.171 | -79.683 |  |
|  |  | β-lactolin | 461.826 | 600.379 | 138.554 | 0.235 |
| DHA | 4-HDHA | Placebo | 10.540 | 9.631 | -0.909 |  |
|  |  | β-lactolin | 10.902 | 15.672 | 4.770 | 0.276 |
|  | 7-HDHA | Placebo | 36.703 | 31.553 | -5.150 |  |
|  |  | β-lactolin | 41.131 | 39.506 | -1.624 | 0.343 |
|  | 14-HDHA | Placebo | 300.402 | 422.831 | 122.428 |  |
|  |  | β-lactolin | 474.837 | 531.392 | 56.555 | 0.672 |
|  | 17-HDHA | Placebo | 58.627 | 48.493 | -10.135 |  |
|  |  | β-lactolin | 74.488 | 73.558 | -0.930 | 0.736 |
|  | DHA | Placebo | 2246.788 | 1984.083 | -262.704 |  |
|  |  | β-lactolin | 2880.569 | 3566.645 | 686.075 | 0.257 |
| rinole acid | HYA+HYC | Placebo | 63.662 | 62.403 | -1.259 |  |
|  |  | β-lactolin | 127.997 | 110.525 | -17.473 | 0.314 |
|  | HYB | Placebo | 171.710 | 166.301 | -5.409 |  |
|  |  | β-lactolin | 220.715 | 198.772 | -21.942 | 0.627 |
|  | KetoA | Placebo | 72.124 | 65.525 | -6.600 |  |
|  |  | β-lactolin | 84.268 | 80.229 | -4.039 | 0.836 |
|  | KetoB | Placebo | 256.445 | 247.556 | -8.889 |  |
|  |  | β-lactolin | 421.452 | 609.265 | 187.814 | 0.438 |

Data are presented as means ± SD. The *p* value shows the between-group difference performed using unpaired *t* tests. β-lactolin group (n = 28); placebo group (n = 28).

**Supplementary Table 5. Changes in microbiome phylum composition in subgroup analysis by age**

|  |  | Age < 54.5 (n = 28) | | | 54.5 < Age (n = 28) | | |
| --- | --- | --- | --- | --- | --- | --- | --- |
|  |  | Baseline (Week 0) | Week 6 | Changes from baseline | Baseline (Week 0) | Week 6 | Changes from baseline |
| Firmicutes | Placebo | 62.94 ± 14.15 | 62.77 ± 15.94 | -0.17 ± 10.47 | 58.43 ± 9.74 | 59.70 ± 12.64 | 1.27 ± 9.91 |
|  | β-lactolin | 53.52 ± 12.97 | 57.24 ± 17.33 | 3.72 ± 13.84 | 58.43 ± 9.74 | 55.90 ± 11.58 | 3.86 ± 10.10 |
|  | p value | 0.078 | 0.388 | 0.409 | 0.177 | 0.414 | 0.499 |
| Actinobacteria | Placebo | 20.45 ± 14.04 | 20.74 ± 13.02 | 0.28 ± 10.72 | 21.76 ± 11.64 | 20.26 ± 12.58 | -1.50 ± 13.31 |
|  | β-lactolin | 26.19 ± 14.7 | 19.28 ± 15.32 | -6.91 ±7.40 | 28.27 ± 15.49 | 20.65 ± 11.21 | -7.63 ± 8.38 |
|  | p value | 0.301 | 0.788 | 0.049 | 0.220 | 0.932 | 0.157 |
| Bacteroidetes | Placebo | 3.12 ± 2.52 | 5.71 ± 5.14 | 2.58 ± 4.84 | 6.26 ± 6.53 | 5.38 ± 4.03 | -0.87 ± 5.73 |
|  | β-lactolin | 5.94 ± 5.98 | 8.06 ± 5.94 | 2.12 ± 6.57 | 5.28 ± 6.03 | 8.46 ± 7.82 | 3.18 ± 5.48 |
|  | p value | 0.117 | 0.273 | 0.837 | 0.684 | 0.578 | 0.067 |
| Proteobacteria | Placebo | 2.76 ± 6.86 | 1.27 ± 2.47 | -1.49 ± 4.47 | 1.82 ± 3.56 | 0.65 ± 0.63 | -1.17 ± 3.55 |
|  | β-lactolin | 0.48 ± 0.65 | 0.55 ± 1.11 | 0.07 ± 0.71 | 1.61 ± 4.03 | 2.11 ± 4.66 | 0.49 ± 3.49 |
|  | p value | 0.226 | 0.328 | 0.209 | 0.886 | 0.257 | 0.222 |
| Verrucomicrobia | Placebo | 0.06 ± 0.13 | 0.07 ± 0.13 | 0.01 ± 0.12 | 0.31 ± 0.53 | 0.45 ± 1.09 | 0.14 ± 1.01 |
|  | β-lactolin | 0.66 ± 1.83 | 0.24 ± 0.39 | -0.42 ± 1.86 | 0.01 ± 0.03 | 0.01 ± 0.02 | 0.00 ± 0.03 |
|  | p value | 0.237 | 0.139 | 0.397 | 0.044 | 0.146 | 0.607 |

Data are presented as means ± SD. The *p* value shows the between-group difference performed using unpaired *t* tests. β-lactolin group (n = 14); placebo group (n = 14).

**Supplementary Table 6. Changes in microbiome genus composition in subgroup analysis by age**

|  |  | Age < 54.5 (n = 28) | | | 54.5 < Age (n = 28) | | |
| --- | --- | --- | --- | --- | --- | --- | --- |
|  |  | Baseline (Week 0) | Week 6 | Changes from baseline | Baseline (Week 0) | Week 6 | Changes from baseline |
| Bifidobacterium | Placebo | 9.84 ± 7.92 | 12.12 ± 9.46 | 2.29 ± 8.17 | 12.85 ± 10.27 | 12.04 ± 8.88 | -0.81 ± 10.89 |
|  | β-lactolin | 17.35 ± 13.02 | 11.54 ± 13.41 | -5.81 ± 8.44 | 19.42 ± 15.2 | 13.09 ± 11.29 | -6.33 ± 7.33 |
|  | p value | 0.077 | 0.896 | 0.016# | 0.192 | 0.785 | 0.128 |
| Bacteroides | Placebo | 1.91 ± 2.07 | 3.87 ± 4.84 | 1.96 ± 4.77 | 4.01 ± 3.78 | 3.64 ± 3.14 | -0.37 ± 3.34 |
|  | β-lactolin | 3.18 ± 3.21 | 4.95 ± 4.22 | 1.77 ± 5.89 | 3.57 ± 4.85 | 6.96 ± 7.12 | 3.39 ± 4.48 |
|  | p value | 0.225 | 0.281 | 0.926 | 0.792 | 0.122 | 0.018# |
| Romboutsia | Placebo | 0.83 ± 1.24 | 0.53 ± 0.45 | -0.30 ± 1.17 | 0.78 ± 2.06 | 0.85 ± 1.41 | 0.07 ± 2.16 |
|  | β-lactolin | 0.68 ± 1.01 | 0.84 ± 1.62 | 0.16 ± 1.36 | 1.16 ± 1.06 | 0.84 ± 1.17 | -0.32 ± 1.19 |
|  | p value | 0.729 | 0.491 | 0.344 | 0.543 | 0.985 | 0.558 |
| Clostridium | Placebo | 0.74 ± 2.17 | 1.63 ± 3.92 | 0.89 ± 3.24 | 0.20 ± 0.35 | 0.73 ± 1.09 | 0.53 ± 0.85 |
|  | β-lactolin | 0.45 ± 0.67 | 0.15 ± 0.31 | -0.30 ± 0.73 | 0.14 ± 0.18 | 0.27 ± 0.51 | 0.13 ± 0.53 |
|  | p value | 0.645 | 0.171 | 0.190 | 0.592 | 0.163 | 0.144 |
| Lactobacillus | Placebo | 0.22 ± 0.74 | 0.60 ± 1.82 | 0.38 ± 1.10 | 0.34 ± 0.78 | 1.17 ± 3.10 | 0.84 ± 2.38 |
|  | β-lactolin | 0.21 ± 0.64 | 0.16 ± 0.30 | -0.05 ± 0.47 | 0.49 ± 1.02 | 0.52 ± 0.80 | 0.03 ± 0.68 |
|  | p value | 0.965 | 0.381 | 0.191 | 0.663 | 0.453 | 0.236 |
| Lactococcus | Placebo | 0.07 ± 0.16 | 0.02 ± 0.03 | -0.05 ± 0.15 | 0.01 ± 0.02 | 0.05 ± 0.14 | 0.04 ± 0.15 |
|  | β-lactolin | 0.01 ± 0.01 | 0.02 ± 0.04 | 0.01 ± 0.04 | 0.01 ± 0.02 | 0.01 ± 0.02 | 0.00 ± 0.04 |
|  | p value | 0.185 | 0.850 | 0.168 | 0.883 | 0.288 | 0.324 |

Data are presented as means ± SD. The *p* value shows the between-group difference performed using unpaired *t* tests. β-lactolin group (n = 14); placebo group (n = 14). #*p* < 0.05 using unpaired *t* tests
